# Effects of *Poria cocos* extract on metabolic dysfunction-associated fatty liver disease *via* the FXR/PPARα-SREBPs pathway

**DOI:** 10.3389/fphar.2022.1007274

**Published:** 2022-10-05

**Authors:** Jinbiao He, Yu Yang, Fan Zhang, Yanjuan Li, Xiaosi Li, Xuemei Pu, Xudong He, Mei Zhang, Xinxing Yang, Qiuman Yu, Yan Qi, Xuefang Li, Jie Yu

**Affiliations:** College of Pharmaceutical Science, School of Clinical Medicine, Yunnan Key Laboratory of Southern Medicinal Utilization, Yunnan University of Chinese Medicine, Kunming, Yunnan, China

**Keywords:** MAFLD (metabolic-associated fatty liver disease), Poria cocos (Schw.) Wolf., bile acid metabolism, FXR/PPARα-SREBP pathway, lipid homeostasis, UPLC Q-TOF/MS

## Abstract

Despite the increase in the global prevalence of metabolic dysfunction-associated fatty liver disease (MAFLD), no approved drug currently exists for the disease. *Poria cocos* (Schw.) Wolf (*P. cocos*) is a medicinal mushroom belonging to a family of polyporaceae widely used in TCM clinics to protect the liver and treat obesity. However, its efficacy, practical components, and underlying mechanism against MAFLD are yet to be determined. In this study, we evaluated the effects of *Poria cocos (P. cocos)* ethanol extract (EPC) on hepatic dyslipidemia, steatosis, and inflammation by both bioinformatics analysis and MAFLD rats induced by HFD feeding. We found EPC treatment dramatically reduced lipid accumulation, inflammatory cell infiltration, and liver injury. EPC reduced serum TC, TG levels, and hepatic TG, TBA, and NEFA contents. UHPLC Q-Trap/MS examination of BA profiles in serum and feces showed that EPC increased fecal conjugated BAs, decreased free BAs, and improved BA metabolism in HFD-fed rats. Western blot and RT-qPCR analysis showed that EPC could activate hepatic FXR and PPARα expression and reduce CYP7A1 and SREBP-1c expression. Systemic pharmacology combined with molecular docking suggested that poricoic acid B and polyporenic acid C, the major active compounds in EPC, could ameliorate lipid homeostasis by activating the nuclear receptor PPARα. We further confirmed their inhibition effects of lipid droplet deposition in steatized L-02 hepatocytes. In summary, EPC alleviated HFD-induced MAFLD by regulating lipid homeostasis and BA metabolism *via* the FXR/PPARα-SREBPs signaling pathway. *P. cocos* triterpenes, such as poricoic acid B and polyporenic acid C, were the characteristic substances of *P. cocos* for the treatment of MAFLD.

## Introduction

Metabolic dysfunction-associated fatty liver disease (MAFLD), formerly known as non-alcoholic fatty liver disease (NAFLD) (Wong and Lazarus, 2021), is the most common liver disease worldwide, affecting 25% of the global population (Younossi et al., 2016). In Asia, the prevalence of MAFLD is projected to increase to 20–35% over the next 10 years (Eslam et al., 2020). It is strongly associated with metabolic disorders, such as obesity, hypertension, dyslipidemia, and type 2 diabetes mellitus (de Alwis and Day, 2008). MAFLD is characterized by excessive accumulation of lipids in the liver, called steatosis (Byrne and Targher, 2015), which can develop into non-alcoholic fatty liver hepatitis, fibrosis, cirrhosis, and hepatocellular carcinoma, ultimately leading to necrosis (Michelotti et al., 2013; Zhu et al., 2016). At present, the MAFLD therapeutic targets are mainly focused on nuclear receptor agonists involved in steatosis, inflammation, or fibrogenesis, such as the farnesoid X receptor (FXR, NR1H4), peroxisome proliferator-activated receptor α (PPARα, NR1C1), as well as analogs of enterohepatic hormones, including fibroblast growth factor (FGF)19 in humans, FGF15 in rodents and FGF21 (Michelotti et al., 2013). Due to the complex pathogenesis of MAFLD, the critical regulatory issues and valid drug targets are unclear. Therefore, developing effective and safe drugs to prevent and treat MAFLD has become an essential issue in the global medical field.

When the rate of lipid influx and ab initio synthesis exceeds the rate of lipid oxidation and release, excess lipids accumulate in hepatocytes, leading to the development of MAFLD (Machado and Diehl, 2016). Hence, a central pathological characteristic of MAFLD is the accumulation of excess TG in the hepatic (de Alwis and Day, 2008). Recent research has shown that promoting *PPARα* gene expression can inhibit the expression of TG synthesis gene *HMGCR* and *SREBP-1c*, thereby reducing lipid accumulation in the hepatic and suppressing MAFLD in mice (Wu et al., 2019). In addition, activation of FXR inhibits the BA rate-limiting enzyme CYP7A1 and the TC synthesis rate-limiting enzyme *SREBP-1c* to regulate BA synthesis, reduce hepatic lipogenesis, and inhibit inflammatory and fibrotic responses to treat MAFLD (Moris et al., 2017; Jiao et al., 2022).

*Poria cocos* (Schw.) Wolf (*P. cocos*) is a medicinal mushroom belonging to a family of polyporaceae, also called Fuling, Tuckahoe, Hoelen, or Indian bread (Lindner and Banik, 2008), is a trophozoite mushroom found in the wilting skins and roots of pine trees in China, Korea, Japan, and North America (Ríos, 2011). In Asian medicine, it has been widely employed as a component of many preparations and as a medicinal and edible species (Lindner and Banik, 2008; Ríos, 2011). According to the ancient Chinese medical masterpieces “Sheng Nong’s herbal classic (AD220‒280),” “Jin Kui Yao Lue (AD198‒201),” “Tai Ping Hui Min He Ji Ju Fang (AD1208)” and “Shang Han Lun (AD198.201),” *P. cocos* has been used in TCM for more than 2,000 years with multiple functions (Esteban, 2009; Li et al., 2019; Nie et al., 2020). Because of its liver-protective, anti-inflammatory, anti-oxidant, and anti-diabetic effects, *P. cocos* has been widely used to treat insomnia, neurological disorders, chronic edema, kidney disease, and heart disease. It has been described as “nine out of ten prescriptions (Ding et al., 2019). The chemical constituents of *P. cocos* are primarily triterpenoids, polysaccharides, and steroids (Qian et al., 2018). Research on chemical composition shows that the foremost effective compounds of *P. cocos* triterpenes are pachymic acid, dehydropachymic acid, polyporenic acid C, dehydrotrametenolic acid, poricoic acid A, poricoic acid B, and so on (Tian et al., 2019). Recent studies have demonstrated that *P. cocos* may alleviate hyperlipidemia and obesity, and *P. cocos* triterpenes have anti-inflammatory, anti-oxidant, diuretic, and hepatoprotective effects (Miao et al., 2016). Both eburicoic acid and dehydroeburicoic acid protect the hepatic against CCl_4_-induced liver injury by anti-oxidant and pro-inflammatory mechanisms (Huang et al., 2013). However, whether *P. cocos* prevents MAFLD and its underlying material basis and mechanism are unclear.

This study aimed to ascertain the effects of *P. cocos* on the etiopathogenesis of MAFLD, including its impact on lipid heterogeneity, lipid accumulation, hepatic damage, and inflammation. Based on the unclear relationship between *P. cocos* on MAFLD, we used the TCM network pharmacology analysis system to establish the gene co-association between *P. cocos* and MAFLD. Subsequently, we evaluated the effects of *P. cocos* ethanol extract (EPC) on hepatic dyslipidemia, steatosis, BA metabolism, and inflammation in MAFLD rats induced by HFD feeding. And finally, the virtual screening combined with UPLC Q-TOF/MS technology and *in vitro* experiments hypothesized that *P. cocos* triterpenes, such as poricoic acid B and polyporenic acid C, were the characteristic substances of *P. cocos* for the treatment of MAFLD. Hence, we explored the pharmacological effects and mechanism of *P. cocos* from lipid homeostasis and BA metabolism through the FXR/PPARα-SREBPs signaling pathway in rats with high-fat diet (HFD)-induced MAFLD.

## Materials and methods

### Reagents

The HFD containing 1% cholesterol, 10% refined lard, 10% egg yolk, and 79% basic feed was provided by Beijing Keaoxieli Feed Co. Ltd. (Beijing, China). The control diet consisted only of basic feed. Fenofibrate capsules (FC) were purchased from Abbott Laboratories (Chicago, IL, United States). The routine biochemical kits were obtained from Jiancheng Bioengineering Institute (Nanjing, China). Enzyme-linked immunosorbent assay (ELISA) kits for rat TBA, TNF-α, IL-1β, IL-6, and Insulin Receptor (IR) were purchased from Jiangsu Zeyu Biotechnology Co., Ltd., (Jiangsu, China, 31200). A sodium dodecyl-sulfate polyacrylamide gel electrophoresis matching kit (cat. no. P0012A) and the BCA protein assay kit (cat. no. P0012) were purchased from Beyotime Biotech Inc. (Shanghai, China). Primers were designed by General Biotech Co. Ltd. (Shanghai, China). Total ribonucleic acid (RNA) extraction kits (cat. no. DP419) were obtained from the Tiangen Biochemical Department, Technology Co., Ltd. (Beijing, China). TaKaRa PrimeScript RT Master Mix (cat. no. RR036A), and TaKaRa TB Green Premix Ex Taq II (cat. no. RR820A) was purchased from Bao Biological Engineering Co. (Dalian, China).

Anti-SCD1 (cat. no. ab9535) and anti-p-NF-κB (cat. no. ab6302) were purchased from Abcam (Cambridge, United Kingdom). Anti-CYP19A1 (cat. no. DF6884), anti-CYP7A1 (DF2612), anti-FGF15 (cat. no. DF2651), anti-PPARα (cat. no. AF5301), anti-p-AMPK (cat. no. AF3423), and anti-SHP1 (cat. no. AF3244) was purchased from Affinity Biosciences (Shanghai, China). Anti-FXR1 (cat. no. 13194-1-AP), anti-NR3C1 (cat. no. 24050-1-AP), anti- fatty acid synthase (FASN) (cat. no. 10624-2-AP), anti-AMPKα (cat. no. 10929-2-AP), anti-NF-κB (cat. no. 10745-1-AP), anti-ERK1/2 (cat. no. 51068-1-AP), anti-p-ERK1/2 (cat. no. 28733-1-AP), anti-JNK (cat. no. 24164-1-AP), anti-p-JNK (cat. no. 80024-1-RP) were purchased from Proteintech (Wuhan, China). Anti-β-actin (cat. no. YT0099) was purchased from ImmunoWay Biotechnology Company (Beijing, China). IRDye^®^680RD Goat anti-Rabbia IgG Secondary Antibody (cat. no. 926-68071) as purchased from LI-COR Biotechnology (Hong Kong, China).

### Medicinal material identification, extraction, and measurement

The identification of *P. cocos*. *P. cocos* was purchased from Puer, Yunnan, China. The samples were authenticated by Professor Jie Yu (Professor at the Yunnan University of Chinese Medicine), and voucher specimens were deposited in the Key Laboratory of Preventing Metabolic Diseases of TCM, Yunnan University of Chinese Medicine (Kunming, China).

Preparation and content determination of EPC. Taking 30 Kg of *P. cocos* add 3 times the volume of 75% ethanol, boiled 2 h, and filtered. Added 2 times the volume of 75% ethanol to the filtered residue, continued to boil for 2 h and filtered. The filtrates were combined twice, filtered, and then concentrated under reduced pressure at 65°C using a rotary evaporator. The extract was frozen at −80°C, then transferred to a lyophilizer and lyophilized to obtain 537 g of EPC. Taking 100 mg of EPC, adding 75% ethanol to 25 ml volumetric flask, and taking 1 ml of solution into 100 ml volumetric flask, using vanillin-glacial acetic acid color displayed method to detect the purity of *P. cocos* triterpenes, its purity is calculated as 57.43%.

### Network pharmacological analysis

Co-associations between MAFLD genes and *P. cocos* targets were analyzed in the TCMNPAS (http://54.223.75.62:3838/) by entering MAFLD (MCID: NNL005) and *P. cocos*, then selecting the HIT, TCMID, STITCH, and TCMSP databases. The drug activity index QED was set to 0.2, the drug association threshold was 400, and the compound target significance was *p* < 0.05 (Ma et al., 2021). Next, based on the absorption, distribution, metabolism, and excretion (ADME) and with the oral availability (OB) set to ≥ 30% and drug similarity (DL) ≥ 0.18, the active components and targets of *P. cocos* were retrieved from the TCMSP database (http://tcmspw.com/index.php). Supplement and back-predicted active ingredients were not found for the target using the PharmMapper database search. (http://www.lilab-ecust.cn/pharmmapper/). The targets corresponding to each active component were corrected using the Uniport Database (http://www.uniprot.org/). Meanwhile, “MAFLD” was entered into the GeneCards website (https://www.genecards.org/), OMIM (https://omim.org/), and DrugBank databases (https://https://go.drugbank.com/) as a search term to collect related target information. Duplicate values were merged and deleted. The targets of the active components of *P. cocos* and MAFLD-related targets were introduced into venny 2.1.0 on the website (https://bioinfogp.cnb.csic.es/tools/venny/), and the intersection was regarded as the target protein of *P. cocos* in treating MAFLD. In addition, the protein-protein interaction network was constructed using the STRING database (https://string-db.org/), minimum interaction (score = 0.4), and the associated interaction network was visualized by Cytoscape 3.8.2 software. Finally, pathways associated with the predicted genes were annotated using DAVID (https://david.ncifcrf.gov/), and gene ontology-cellular component (GO-CC), Gene ontology-biological process (GO-BP), Gene ontology-molecular function (GO-MF), and Kyoto Encyclopedia of Genes and Genomes (KEGG) enrichment analyses were carried out (*p* ≤ 0.0001, count >5) and exported.

### Target organ analysis

The key genes determined to be core targets of *P. cocos* in the treatment of MAFLD were imported into the BioGPS database (http://biogps.org/). Statistical analyses were then used on each result to determine the target organs by setting the target to a level higher than the average score. Cytoscape 3.8.2 was employed to construct the target-organ localization direct relationship network.

### *In vivo* experimental design

Sprague–Dawley male rats (220 ± 20 g) were purchased from Dashuo Biotech Co. (SLAC, Hunan, China). All the rats were maintained in a pathogen-free environment with controlled conditions of 24 ± 2°C, 45 ± 10% humidity, and a 12 h light/dark cycle. Rats were acclimated to the environment on a control chow diet administered *ad libitum* for 1 week and were then randomly divided into five groups (*n* = 10 per group) as follows: 1) normal diet control group (CON, containing 10% kcal fat), 2) high-fat diet group (MOD, containing 60% kcal fat), 3) 20 mg/kg FC group, 4) low dose (56.3 mg/kg) and 5) high dose (168.9 mg/kg) of EPC group (EPC-L and EPC-H). The dosage of EPC-L used in rat experiments was calculated according to the known *P. cocos* dose of humans (10 g/day/person), which was recorded in the Chinese Pharmacopoeia (2020). Rats were treated daily from the first week to the end of the experiment (which lasted 12 weeks). The food intake of all the rats was recorded daily, and body weights were recorded weekly. At the end of this experiment, the rats were anesthetized using 1% pentobarbital sodium. Samples of blood, heart, liver, lung and adipose tissues were collected for hematoxylin and eosin (H&E) staining or liquid nitrogen flash freezing, followed by refrigerator storage at −80°C for later analysis.The liver tissue was fixed with 4% paraformaldehyde for 24 h and then sectioned by dehydration, embedded in paraffin, cut into 5–7 μm sections, stained with H&E, and examined under a light microscope (Thermo, Waltham, MA, United States) at 200× magnification. Five animals in each group were observed under the microscope in four fields of view, for changes in liver tissue.

### Determination of serum and liver biochemical parameters

Serum samples (200 μL) were analyzed using a biochemical analyzer (Beckman CX4, Roche, Germany) to measure the levels of triglyceride (TG), total cholesterol (TC), high-density lipoprotein cholesterol (HDL-C), Low-density lipoprotein cholesterol (LDL-C), aspartate aminotransferase (AST), alanine aminotransferase (ALT), and glucose (GLU). Precisely 80 mg of rat liver tissue was weighed and placed into 720 µL of physiological saline, then homogenized. The supernatant was collected by centrifugation at 12004 *×g* for 15 min at 4°C, and the protein concentration was determined using BCA kits. Determination of TG, TC, LDL-C, HDL-C, AST, ALT, and nonesterified free fatty acids (NEFA) levels in the liver was performed according to the standard operating instructions of the kit.

### Measurement of inflammatory factors

Precisely 80 mg of rat liver tissue was weighed and placed into 720 µL of phosphate-buffered saline buffer (pH 7.4), then homogenized. The supernatant was collected by centrifugation at 12004 *×g* for 15 min at 4°C, and the protein concentration was determined using BCA kits. According to the manufacturer’s protocol, total bile acid (TBA), indirect bilirubin (IBIL), insulin receptor (IR), interleukin -1β (IL-1β), interleukin -6 (IL-6), and tumor necrosis factor-α (TNF-α) were quantified by ELISA kits.

### Quantitative analysis of serum and ileal fecal BAs

50 mg were accurately weighted, and then add 400 μL of extraction solution (methanol: water = 4:1), grind at 4°C for 6 min, then grind with a freezer grnder for 6 min (−10°C, 50 Hz). An ultrasound (5°C, 40 kHz) for 30 min, centrifuged at 13000 *×g* for 15 min at 4°C. Finally, the supernatant was injected into the LC-MS/MS system for analysis. 50 μL was accurately weighted, and then add 100 μL methanol by good vortexing for the 30 s, ultrasound (5°C, 40 kHz) for 30 min, standing for 30 min, and centrifuged at 13000 *×g* for 15 min at 4°C. Afterward, 100 μL of supernatant were collected and blown dry with a nitrogen blower. Then the residue was reconstituted with 100 μL 25% acetonitrile solution. Finally, the supernatant (1 μL) was injected into the LC-MS/MS system for analysis. The analysis was performed using an UHPLC Q-Trap/MS for the sample’s qualitative and quantitative detection of the target substances. Analyte compounds were separated with a Waters BEH C18 (150*2.1 mm, 1.7 μm) Liquid chromatography column (AB SCIEX). The mobile phases consisted of 0.1% formic acid—water solution (solvent A) and 0.1% formic acid—acetonitrile solution (solvent B), delivered at a flow rate set to 0.35 ml/min. The solvent gradient changed according to the following conditions: from 0 to 6 min, 31% A to 31% A, 29% B to 29% B; from 6 to 22 min, 31% A to 25% A, 29% B to 75% B; from 22 to 22.1 min, 25% A to 0% A, 75% B to 100% B; from 22.1 to 25 min, 0% A to 0% A, 100% B to 100% B for equilibrating the systems. The column temperature was maintained at 40°C. During the period of analysis, all these samples were stored at 4°C. Mass spectrometry conditions: AB SCIEX QTRAP 6500+ with negative mode detection, Curtain Gas (CUR) of 35, Collision Gas (CAD) is Medium, IonSpray Voltage (IS) is −4500 V, Temperature (TEM) is 500°C, Ion Source Gas1 (GS1) is 40, Ion Source Gas2 (GS2) is 50.

### Real-time quantitative PCR analysis

Total RNA was prepared from livers using Trizol reagent according to the manufacturer’s instructions. RNA was equalized and converted to cDNA using a HiScript II reverse transcriptase kit. Gene expression was measured by RT-qPCR (Roche, Basel, Switzerland) using SYBR Green. A total of 1000 ng of cDNA template was used for PCR amplification using the primers listed in Supplementary Table S1. The total reaction volume was 20 μL, including SYBR Premix Ex TaqII (9 μL), Primer F (0.3 μL), Primer R (0.3 μL), cDNA (2 μL), and ddH_2_O (8.4 μL). The PCR conditions were as follows: pre-denaturation at 95°C for 30 s, denaturation at 95°C for 5 s, annealing and extension for 20 s at 62°C, and amplification for 40 cycles. Glyceraldehyde-3-phosphate dehydrogenase (GAPDH) was used as an internal reference. Experiments were conducted in triplicate for each sample, and the 2^−ΔΔCT^ method was used to determine the relative level of target gene expression.

### Western blot analysis

Precisely 60 mg of rat liver tissue was weighed and placed into 300 µL of radio-immunoprecipitation assay buffer (Beyotime Technology, Shanghai, China) until completely lysed, and the supernatants were collected by centrifugation. The total protein concentration was determined using the BCA protein assay kit. Then, 5 μg/μL of protein was separated by gel electrophoresis and transferred onto a polyvinylidene difluoride membrane. The membrane was blocked with 5% skim milk for 2 h at 20−25 °C and then incubated with primary antibodies anti-CYP19A1 (1:1000), anti-CYP7A1 (1:1000), anti-FASN (1:2000), anti-SCD1 (1:1000), anti-FXR (1:1000), anti-FGF15 (1:1000), anti-SHP1 (1:1000), anti-AMPKα (1:2000), anti-p-AMPK (1:1000), anti-NR3C1 (1:4000), anti-JNK1 (1:5000), anti-p-JNK (1:2000), anti-ERK (1:1000), anti-p-ERK (1:5000), anti-NF-κB (1:2000), anti-p-NF-κB (1:1000), and anti-PPARα (1:1000) overnight at 4°C. Membranes were then washed and incubated with IRDye 680RD Goat anti-Rabbit for 2 h at 20–25°C, and the signal was detected using an enhanced immunofluorescence substrate. Immunoreactive bands were quantified using the ImageJ software.

### Molecular docking analysis

Poricoic acid C, polyporenic acid C, poricoic acid B, dehydroeburicoic acid, eburicoic acid, dehydrotumulosic acid, (22E)-ergosta-7, dehydropachymic acid, poricoic acid A, 16 alpha-hydroxydehydrotrametenolic acid, and 22-diene-3β-ol were used as ligands. FXR, TNF, PPARα, CYP19A1, PPARγ, NR1H3, RXRα, HMGCR, FASN, and NR3C1 were used as protein receptors. The PubChem database (https://pubchem.ncbi.nlm.nih.gov/) was used to download ligands’ two-dimensional (2D) structures. It was processed and transformed into PDB format through Chem3D (Chem3D 18.0.0.231) and saved in PDBQT format. The X-ray crystal structures of the targets (http://www.rcsb.org/), including TNF (PDB ID: 1FT4), FXR (PDB ID: 6A5G), PPARα (PDB ID: 6L37), CYP19A1 (PDB ID: 4KQ8), PPARγ (PDB ID: 6T6B), NR1H3 (PDB ID: 6AVI), RXRα (PDB ID: 6JNO), HMGCR (PDB ID: 3CD0), FASN (PDB ID: 6NNA), and NR3C1 (PDB ID: 6DXK), were obtained from the PDB database (https://www.pdbus.org/). Subsequently, the protein receptor files were processed and converted to PDBQT format using AutoDock Tools 1.5.6. Finally, Autodock Vina v.1.1.2 was run to perform molecular docking. The conformation with the best affinity was selected as the final docking conformation, and the results of the best binding energy were visualized using PyMOL 2.5.0. Moreover, hydrogen and hydrophobic bonds were analyzed for ligand and receptor binding using the PLIP (https://plip-tool.biotec.tu-dresden.de/).

### Analysis and identification of EPC by UPLC Q-TOF/MS

Identification of the main active ingredients was performed by the Agilent UPLC Q-TOF/MS liquid mass spectrometer (Agilent Technologies, Inc., California, United States). Chromatographic column Agilent Zorbax SB-C18 (50*2.1 mm, 1.8 μm); column temperature 30°C; mobile phase: acetonitrile for A, water for B; flow rate 0.3 mL/min; detection wavelength 203nm; injection volume 5 μL; and running time 70 min. The solvent gradient changed according to the following conditions: from 0 to 5 min, 5% A to 5% A, 90% B to 85% B; from 20 to 30 min, 15% A to 25% A, 895% B to 75% B; from 30 to 40 min, 25% A to 45% A, 75% B to 55% B; from 40 to 50 min, 45% A to 70% A, 55% B to 30% B; from 50 to 60 min, 70% A to 100% A, 30% B to 0% B; from 60 to 70 min,100% A to 100% A, 0% B to 0% B; from 70 to 70.1 min, 100% A to 5% A, 0% B to 95% B. Mass spectrometry with positive and negative ion scan mode detection; ion source was ESI; the temperature was 350°C; collision gas and drying gas flow rate was 8 L/min; capillary voltage: 3.5 kV; atomization pressure: 30 psi; mass range *m/z*: ∼100–1,200 ppm; and collision energy: 10, 20, 30, 40, 50 msec.

### *In vitro* experiments

*In vitro* experiments were performed using L-02 hepatocytes cultured in RPMI 1640 medium at 37 °C with 10% fetal bovine serum, 1% penicillin-streptomycin, and 5% CO_2_. Cells were seeded into 6-well plates at a density of 3 × 10^5^ cells per well; upon reaching 80-90% confluency, they were starved in 0.2% serum for 12 h, exposed to 5% fat emulsion for 24 h, and then cultured with medium containing poricoic acid B (25, 50, and 100 μmol/L), polyporenic acid C (25, 50, and 100 μmol/L), eburicoic acid (25, 50, and 100 μmol/L), and FC (150 μmol/L) or separate medium (CON group) for 24 h and harvested. The levels of TG, TC, AST, and ALT in the cell supernatant were measured according to the manufacturer’s instructions. In addition, the extent of lipid accumulation in L-02 hepatocytes was determined by Oil Red O staining according to the manufacturer’s instructions. Briefly, replicate the hepatic L-02 cell steatosis model after administration of drug stimulation for 24 h. Washed twice with PBS, then added 2 mL of 4% paraformaldehyde per well for 30 min to fix and then aspirate and discard paraformaldehyde. Added 2 mL of 60% isopropanol to each well for 5 min, uniformly added 2 mL of Oil Red O staining working solution slowly along the wall of each well for 35 min, then rinse twice carefully with PBS, then add 1 ml of hematoxylin staining solution slowly along the wall of each well for 2 min, and rinse 3 times with PBS again. The Cx31 Olympus imaging system (Olympus, Tokyo, Japan) was used to capture images.

### Statistical analysis

Statistical analyses were performed using SPSS version 21.0 (IBM, Armonk, NY, United States). Measurement data were expressed as the mean ± standard deviation. Paired *t*-tests were adopted for comparisons of the paired data between two groups with normal distribution and homogeneity of variance, while unpaired *t*-tests were performed for comparisons of unpaired data. One-way analysis of variance (ANOVA) or repeated-measures ANOVA was conducted for multiple group comparisons, followed by Tukey’s post hoc test. Pearson’s correlation was used to analyze the correlation of the observed indexes. A value of *p <* 0.05 was considered statistically significant.

## Result

### Intrinsic association and active compound identification of *P. cocos* for the treatment of metabolic dysfunction-associated fatty liver disease

**FIGURE 1:**
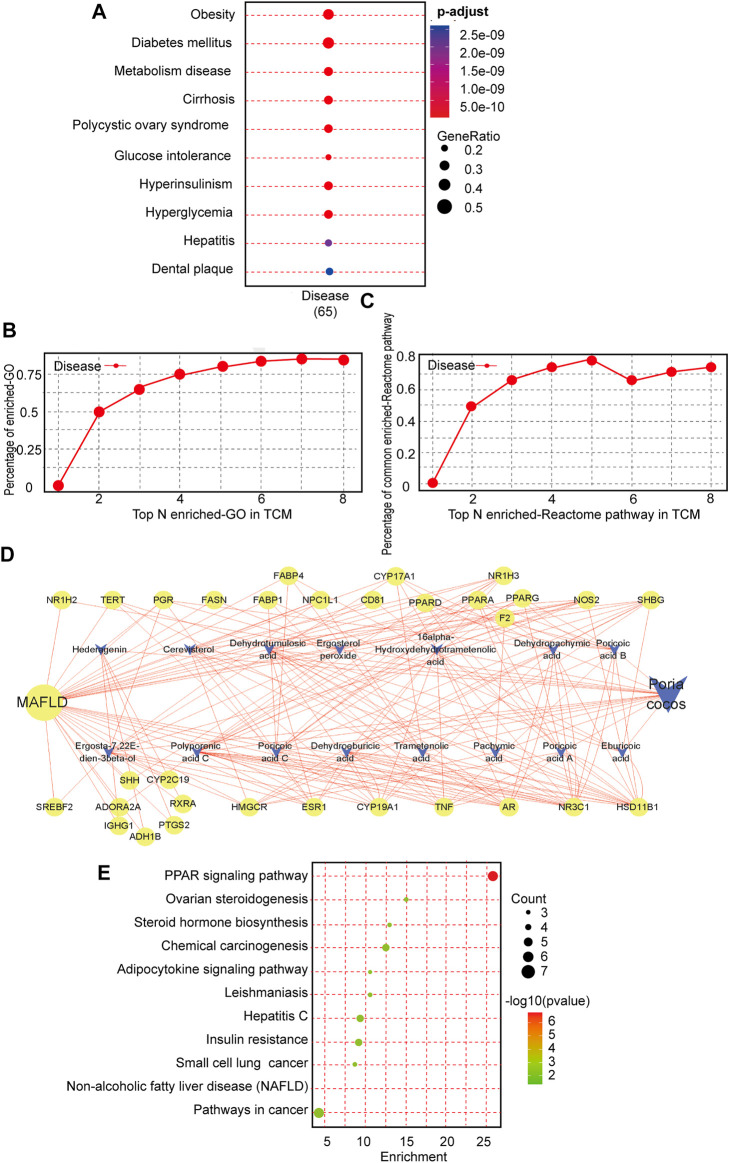
Intrinsic association and active compound identification of *Poria cocos* (*P. cocos*) for the treatment of MAFLD. **(A)** Metabolic dysfunction-associated fatty liver disease (MAFLD) Gene ontology (GO) enrichment. **(B)** Similarity of co-associated GO terms between *P. cocos* and MAFLD. The similarity between the co-associated GO terms of *P. cocos* and MAFLD was 0.87, and the area under the curve (AUC) of the top 30 of these terms was 4.84. **(C)** Similarity of co-associated Reactome pathways between *P. cocos* and MAFLD. The similarity between co-associated Reactome pathways of *P. cocos* and MAFLD was 0.78, and the AUC of the top 30 of these pathways was 4.47. **(D)** MAFLD-*Poria cocos*-target relationship network diagram. Nodes of higher degree value are indicated by a larger diameter and darker color. Stronger interactions between nodes are indicated with thicker edges and darker colors. Green represents the targets, orange represents the compound, and blue represents the disease. The circle represents the target and “V” represents the active component of *P. cocos*.**(E)** The Kyoto encyclopedia of genes and genomes (KEGG) pathway enrichment analysis of key targets.

To investigate whether there is a relationship between *P. cocos* and MAFLD, we used the TCMNPAS database to establish gene co-associations between the MAFLD and *P. cocos*. MAFLD was found to be related mainly to obesity, diabetes mellitus, metabolic diseases, cirrhosis, polycystic ovary syndrome, glucose intolerance, fatty liver, as well as other diseases. This was consistent with the results of clinical and laboratory studies and provided evidence for further questions addressed in this study (Figure 1A). Similarity of co-associated Reactome pathways and GO terms between *P. cocos* and MAFLD is shown in Figures 1B,C.

According to the ADME analysis, 15 main active (Supplementary Table S2) ingredients of *P. cocos* were screened and collated to obtain 125 potential targets. Screening according to a 2-fold degree (DC **≥** 8) revealed that poricoic acid C, polyporenic acid C, poricoic acid B, dehydroeburicoic acid, eburicoic acid, dehydrotumulosic acid, dehydropachymic acid, poricoic acid A, 16 α- hydroxydehydrotrametenolic acid, and (22E)-ergosta-7, 22-diene-3β-ol might be the key active components of *P. cocos* (Figure 1D). The above suggested that the total triterpenes of *P. cocos* may be one of the active ingredients in the treatment of MAFLD**.**

### Enrichment analysis of key pathways and organs

Subsequently, in order to screen the key organs and pathways of *P. cocos* for the treatment of MAFLD. A total of 1397 MAFLD targets were obtained through data filtration, and 31 cross-over genes with *P. cocos* were identified (Supplementary Figures S1A–S1B **)**. The most significant terms in the GO-BP, GO-MF, and GO-CC categories at *p* < 0.0001 with a count >5 are displayed in supplementary material Figure 1C. There are 11 signaling pathways screened by KEGG pathway enrichment analysis (Figure 1E). Among them, the top three signaling pathways are the metabolic signaling pathway, PPAR signaling pathway, and cancer signaling pathway. This suggested that the treatment of MAFLD by *P. cocos* may be related to the PPARα signaling pathway. Furthermore, the 31 intersecting genes of *P. cocos* and MAFLD were entered into the BioGPS database. Taking the above-average level as the screening standard for gene localization in tissues and organs, the main target tissues and organs were the pineal gland, liver, lung, heart, and intestine (Supplementary Figure S1D).

### Effects of EPC on metabolic dysfunction-associated fatty liver disease in HFD-induced rats

**FIGURE 2:**
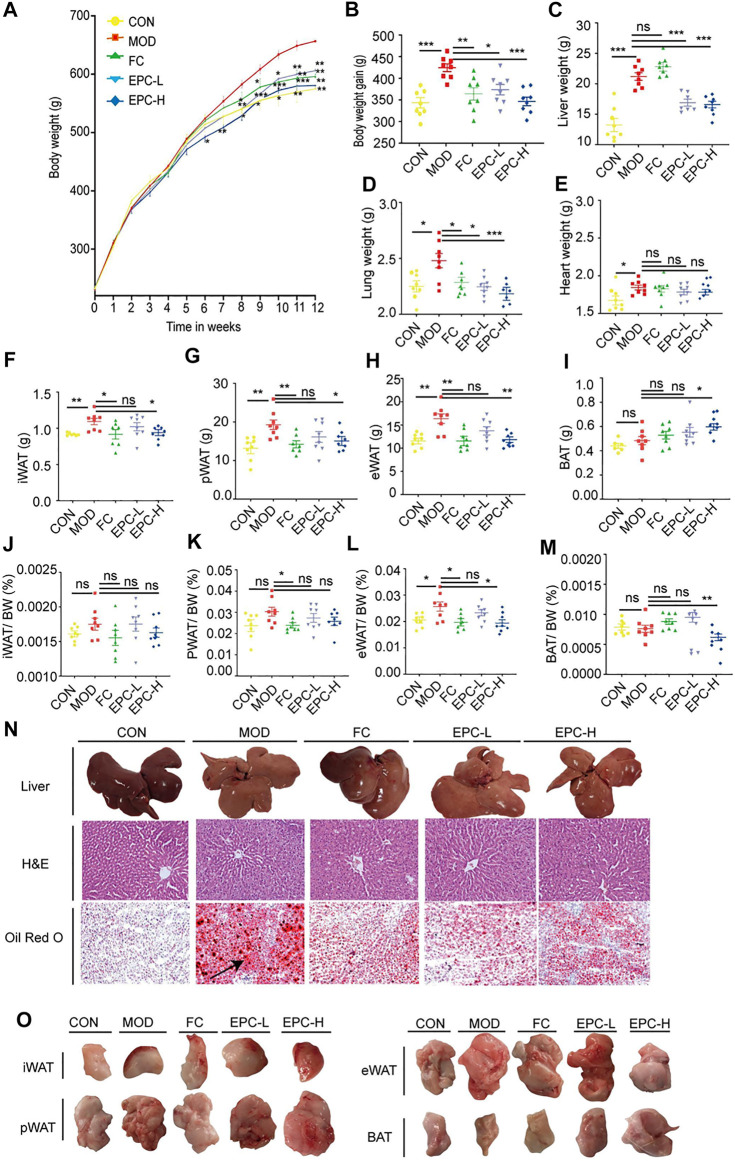
EPC ameliorated MAFLD in rats. **(A)** Body weight (BW). **(B)** BW gain. **(C–E)** Organ wet weight. **(F)** Inguinal white adipose tissue (iWAT). **(G)** Perirenal white adipose tissue (pWAT). **(H)** Epididymis white adipose tissue (eWAT). **(I)** Brown adipose tissue (BAT). **(J)** iWAT/BW ratio. **(K)** pWAT/BW ratio; **(L)** eWAT/BW rati. **(M)** BAT/BW ratio. **(N)** Representative rat liver images of hematoxylin and eosin **(H, E)** and Oil Red Ostaining per group (X200). (O) Representative iWAT, pWAT, eWAT, BAT. One-way analysis of variance (ANOVA) was conducted for the group comparison. n = 8, data are presented as mean ± SEM. *p < 0.05, **p < 0.01, ***p < 0.001 vs. MOD group. EPC, P. cocos ethanol extract; CON, normal diet control group; MOD, high-fat diet group; FC, Fenofibrate capsules; EPC-L, low-dose P. cocos ethanol extract; EPC-H, high-dose P. cocos ethanol extract.

To investigate the role of EPC in MAFLD, we prepared MAFLD rat models by HFD induction for 12 weeks and simultaneously administered EPC to receive different doses of treatment (Figure 2A). At the end of week 12, we weighed the body, liver, lung, heart and inguinal white adipose tissue (iWAT), perirenal white adipose tissue (pWAT), and epididymal white adipose tissue (eWAT) of the rats. In addition, H&E and Oil Red O staining images of the liver suggested MAFLD rat models were successfully induced in this study.

FC and EPC had significantly lower body weight and weight gain compared to the HFD rats (*p* < 0.01) (Figures 2A,B). Similar to the trend in body weight gain, EPC-L and EPC-H reduced liver weight gain (*p* < 0.001) (Figure 2C). Following the organ localization results, we also weighed the lungs and hearts of the rats and found that EPC-L reduced the wet weight of the lungs (*p* < 0.001), but EPC had no significant effect on the weight of the heart (Figures 2D,E). Treatment with EPC significantly reduced iWAT, pWAT, and eWAT (Figures 2F–H) and markedly increased the content of brown adipose tissue (BAT) (*p* < 0.05) (Figures 2I,O). The iWAT, pWAT, eWAT, and BAT to body weight in the MAFLD rats (*p* < 0.05) are displayed in Figures 2J–M. In addition, H&E and Oil Red O staining showed that FC and EPC-L/H treatment reduced lipid accumulation (Figure 2N). The above results indicated that EPC ameliorated MAFLD formation in HFD-induced rats.

### Effects of EPC on blood lipid levels in rats

**FIGURE 3:**
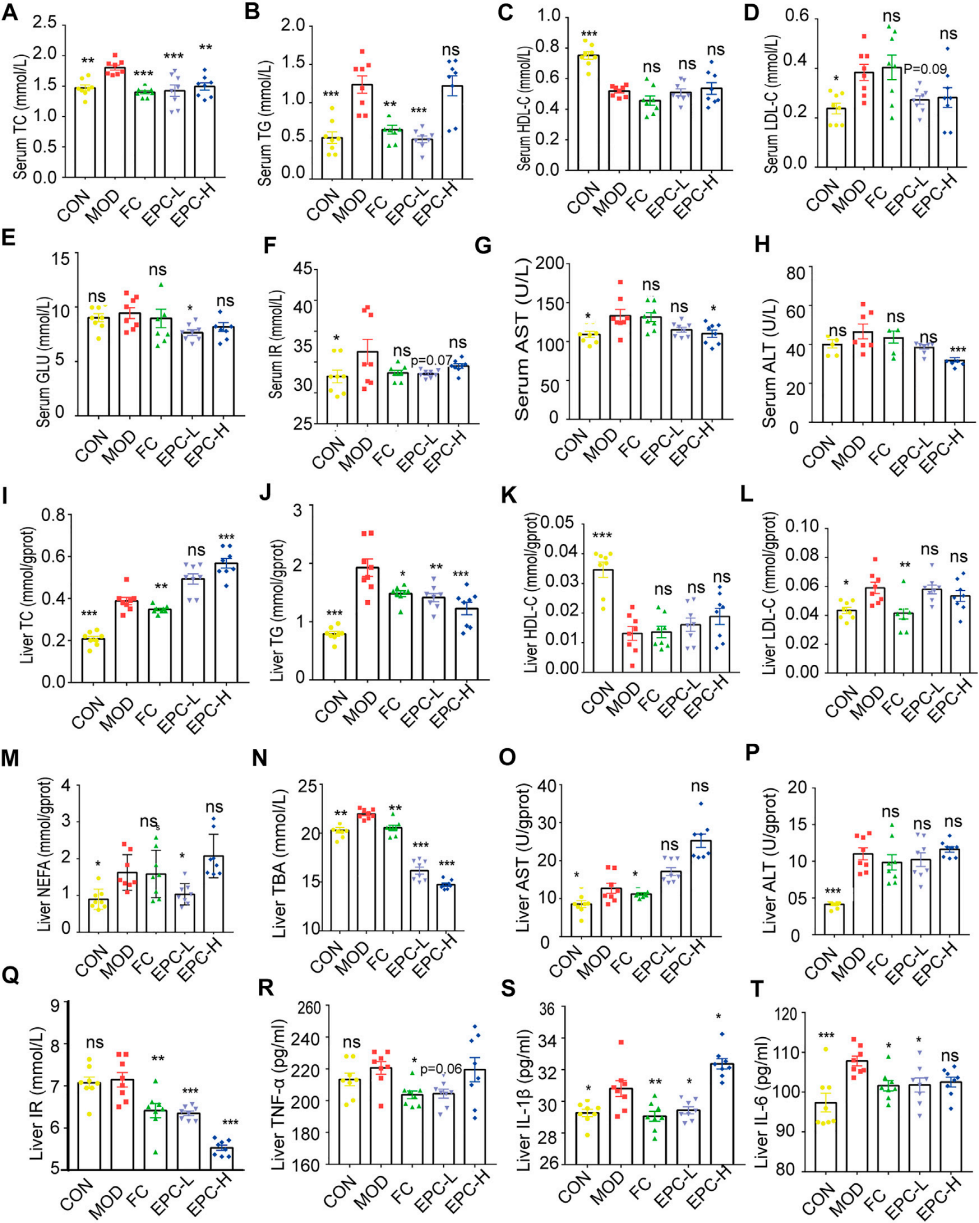
EPC administration improved lipid metabolism in HFD-fed rats. **(A–H)** Serum concentrations of TC, TG, HDL, LDL, GLU, IR, AST, and ALT in the rat. **(I–T)** Liver concentrations of TC, TG, HDL, LDL, NEFA, TBA, AST, ALT, IR, TNF-α, IL-1β, and IL-6. One-way analysis of variance (ANOVA) was conducted for the group comparison. *n* = 8, data are presented as mean ± SEM. *p < 0.05, **p < 0.01, ***p < 0.001 *vs* MOD group. TC, cholesterol; TG, triglyceride; BA, bile acid; TBA, total bile acid; NEFA, nonesterified free fatty acids; ALT, alanine transaminase; AST, aspartate transaminase; NEFA, nonesterified free fatty acid; LDL-C, low-density lipoprotein cholesterol; HDL-C, high-density lipoprotein cholesterol; TNF-α, tumor necrosis factor-α; IL-1β, Interleukin -1β; IL-6, Interleukin -6; IR, Insulin Receptor; GLU, glucose.

To determine the protective effect of EPC on MAFLD, we tested several conventional indicators (Figure 3). The serum levels of TC, TG, LDL, AST, and IR were significantly higher in MAFLD rats. Similarly, the levels of TC, TG, LDL, AST, ALT, NEFA, IR, TNF-α, IL-1β, IL-6, and TBA in their livers were remarkably higher. Moreover, HFD feeding also reduced HDL levels in the liver and serum of rats. Conversely, EPC-L/H can reverse the elevation of TC and TG in serum due to HFD (Figures 3A,B). EPC-H decreased liver and serum TG levels by 30% and 38%, respectively, but did not reduce hepatic TC levels (Figures 3I,J). Also, EPC did not noticeably increase HDL or reduced LDL levels in serum and liver (Figures 3C,D and Figures 3K–L), and the differences in serum AST and ALT levels showed that EPC had a protective effect against liver injury caused by the HFD (Figures 5G,H). Besides, EPC also regulated glucose metabolism disorder caused by the HFD (Figures 3E,F). After the administration of EPC, the NEFA, TBA, TNF-α (*p* = 0.056), IL-1β, and IL-6 levels in rat livers (Figures 3M–T) were significantly decreased. These results suggested that EPC restored abnormal blood lipid levels in HFD-induced rats.

### Effect of EPC on the concentration of BAs in serum and feces of metabolic dysfunction-associated fatty liver disease rats

**FIGURE 4:**
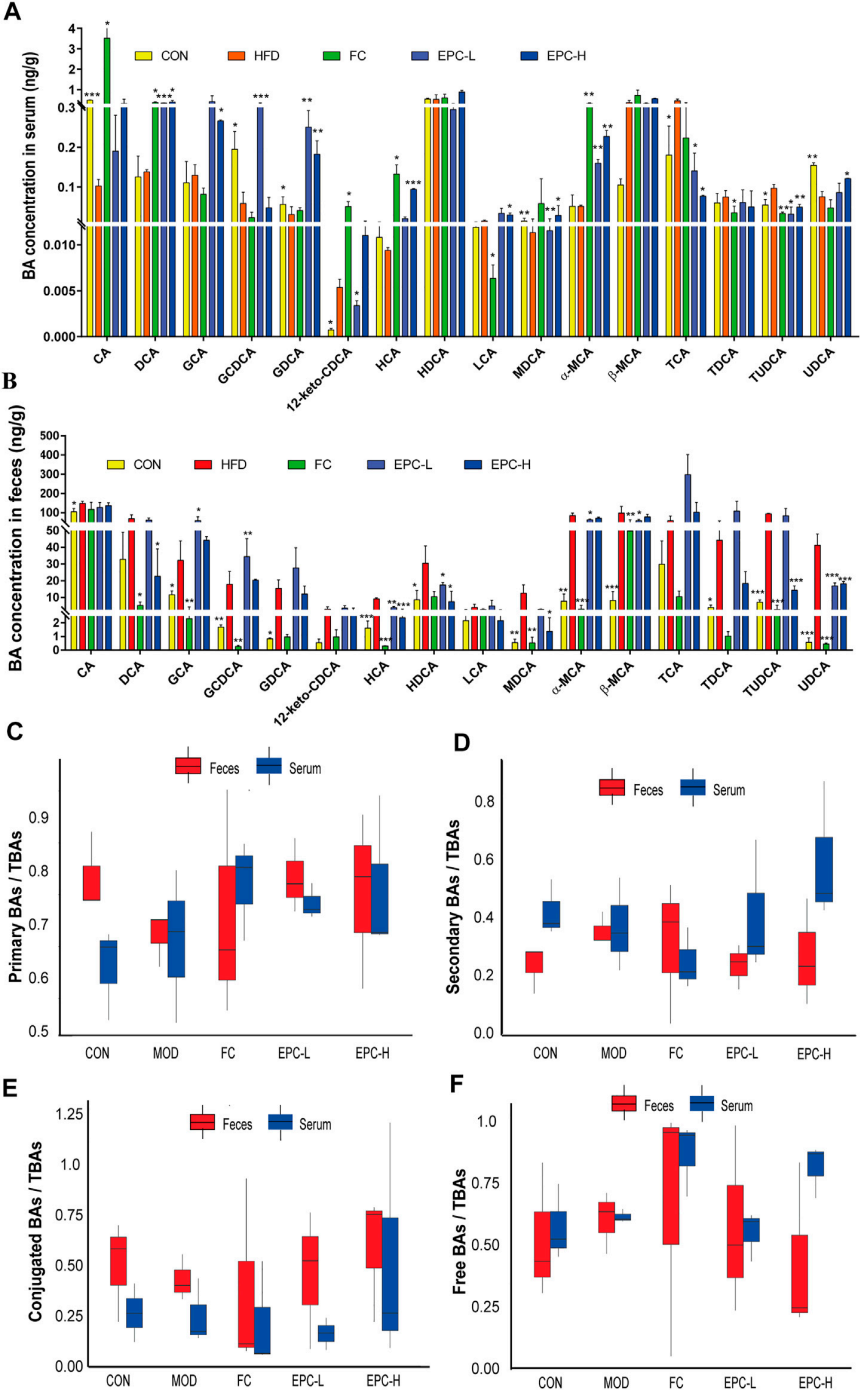
Effect of EPC on bile acid profiles in serum and feces. **(A)** BAs concentration in serum. **(B)** BAs concentration in feces. **(C)** Serum and fecal primary BAs concentration/serum and fecal TBAs concentration box chart. **(D)** Serum and fecal secondary BAs concentration/serum and fecal BAs concentration box chart. **(E)** Serum and fecal concentrations of conjugated BAs/serum and fecal TBAs concentrations box chart. **(F)** Free BAs concentration in serum and feces/serum and fecal BAs concentration box chart. *n* = 3, data are presented as mean ± SEM. CA, 12-keto-CDCA, HDCA, MDCA, and TCA in serum and GDCA, MDCA, HDCA, TDCA in feces by Tukey’s post hoc test. The rest of the data were tested using paired Student‘s *t*-tests. *p < 0.05, **p < 0.01, ***p < 0.001 *vs* MOD group. CA, cholic acid; GCDCA, glycine (G)-chenodeoxycholic acid; GDCA, G-deoxycholic acid; MDCA, murideoxycholic acid; UDCA, ursodeoxycholic acid; TUDCA, taurine (T)-UDCA; HCA, hyocholic acid; HDCA, hyodeoxycholic acid.

To determine the effect of EPC on BAs in MAFLD rats, we used UHPLC Q-Trap/MS to examine the BA profile in serum and feces. The concentrations of cholic acid (CA), glycine (G)-chenodeoxycholic acid (GCDCA), G-deoxycholic acid (GDCA), murideoxycholic acid (MDCA), and ursodeoxycholic acid (UDCA) in serum and ileal feces of MAFLD rats were significantly decreased (Figures 4A,B). Using the ratios of total primary, total secondary, total conjugated, and total free BAs to the corresponding TBAs in serum and feces, we found the EPC-H exhibited higher levels of conjugated BAs, and the EPC-L group showed lower levels of serum-free BAs. Fecal concentrations of primary and conjugated BAs were elevated and secondary BAs, and free BAs were reduced (Figures 4C–E). Specifically, EPC substantially increased serum concentration of deoxycholic acid (DCA), GDCA, muricholic acid (MCA), and α-MCA and dramatically decreased the concentration of taurine (T)-UDCA (TUDCA), and TCA in MAFLD rats (Figure 4A). In addition, the EPC-L notably increased the levels of GCA, GCDCA, and restored the levels of hyocholic acid (HCA), hyodeoxycholic acid (HDCA), α-MCA, β-MCA, and UDCA in ileal feces. EPC-H emphatically reduced the ileum’s DCA, HCA, HDCA, MDCA, and UDCA levels (Figure 4B).

### Effects of EPC on metabolic dysfunction-associated fatty liver disease formation by regulating the expression of genes and proteins related to lipid metabolism

**FIGURE 5:**
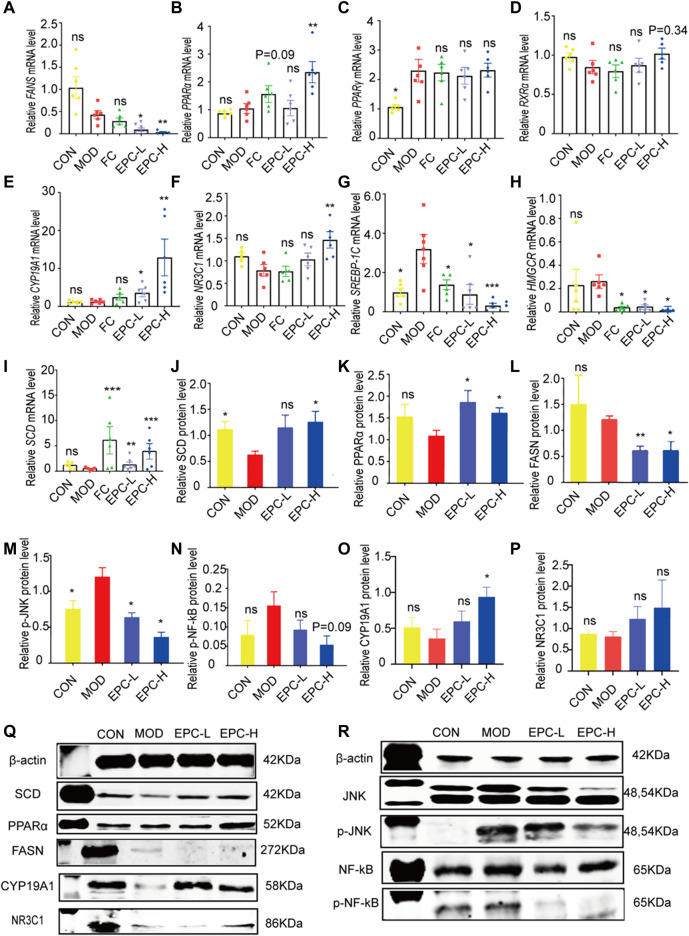
EPC regulated the lipid metabolism-related genes and proteins inMAFLD rats. **(A)** mRNA abundances of *FASN*. **(B)** mRNA abundances of *PPARα (PPARΑ)*. **(C)** mRNA abundances of *PPARγ (PPARG)*. **(D)** mRNA abundances of *RXRα*. **(E)** mRNA abundances of *CYP19A1*. **(F)** mRNA abundances of *NR3C1*. **(G)** mRNA abundances of *SREBP-1c*. **(H)** mRNA abundances of *HMGCR*. **(I)** mRNA abundances of *SCD. n* = 6; **(J)** Relative expression of protein SCD. **(K)** Relative expression of protein PPARα. **(L)** Relative expression of protein FASN. **(M)** Relative expression of protein p-JNK. **(N)** Relative expression of protein p-NF-κB. **(O)** Relative expression of protein CYP19A1. **(P)** Relative expression of protein NR3C1. **(Q-R)** Representative immunoblotting images of β-actin, SCD, PPARα, FASN,CYP19A1, NR3C1, JNK, p-JNK, NF-κB, pNF-κB; *n* = 4, data are presented as mean ± SEM. One-way analysis of variance (ANOVA) was conducted for the group comparison. *p < 0.05, **p < 0.01, ***p < 0.001 *vs* MOD group. FASN, fatty acid synthase; PPARα/γ, peroxisome proliferator-activated receptor alpha/gamma; RXRa, retinoic acid receptor alpha; SREBP-1c, sterol regulatory element binding protein 1c; HMGCR, 3-hydroxy-3-methylglutaryl-coenzyme A reductase; SCD, Stearoyl-CoA desaturase; p-JNK, phosphorylation (p) -stress-activated protein kinase JNK; NF-κB, nuclear factor kappa B.

To explore the mechanism of the action of EPC in lipid metabolism, we examined the expression of several genes and proteins related to lipid metabolism. As shown in Figure 5, administration of EPC prominently increased the expression levels of *CYP19A1*, *NR3C1*, *PPARα* (*p* < 0.01), and *SCD* mRNA and significantly decreased the expression levels of *FASN*, *SREBP-1c*, and *HMGCR* mRNA (Figures 5A–I). Relative to the MOD group, the protein expression levels of CYP19A1, NR3C1, PPARα (*p* < 0.01), and SCD increased markedly (Figures 5J,K,O–P). The FASN (*p* < 0.05), p-JNK*/*JNK (*p <* 0.05), and p-NF-κB/NF-κB(*p* = 0.089) decreased dramatically after EPC treatment (Figures 5L–N).

### Effects of EPC on metabolic dysfunction-associated fatty liver disease formation by regulating the expression of genes and proteins related to BA metabolism

**FIGURE 6:**
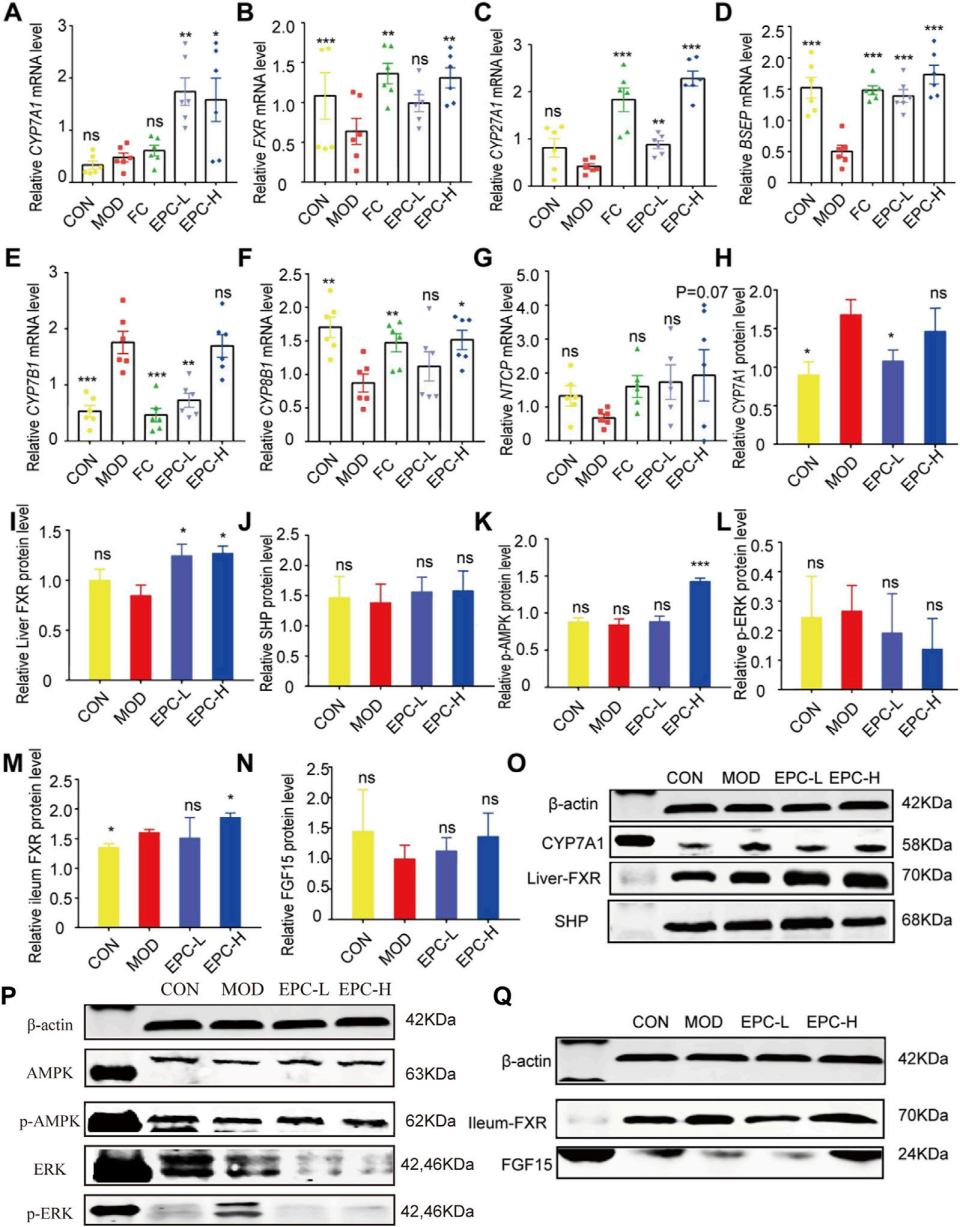
EPC ameliorated MAFLD formation in rats by regulating BA metabolism. **(A–G)** Relative expression of CYP7A1, FXR, CYP27A1, BSEP, CYP7B1, CYP8B1, NTCP mRNA in liver, n = 6; **(H–L)** Relative expression of protein CYP7A1, FXR, SHP, p-AMPK, and p-ERK in the liver, n = 4; **(M–N)** Relative expression of protein FXR and FGF15 in the ileum, n = 4. **(O–P)** Representative immunoblotting images of CYP7A1, FXR, SHP, p-AMPK,and p-ERK in the liver. **(Q)** Representative immunoblotting images of FXR and FGF15 in the ileum. Data are presented as mean ± SEM. One-way analysis of variance (ANOVA) was conducted for the group comparison. *p < 0.05, **p < 0.01, ***p < 0.001 vs. MOD group. CYP7A1, cholesterol 7α-hydroxylase; FXR, farnesoid X receptor; CYP27A1, sterol 27-hydroxylase; BSEP, bile salt export protein; CYP7B1, oxysterol 7α-hydroxylase; CYP8B1, sterol 12αhydroxylase; NTCP, Na + -taurocholate co-transporting polypeptides; SHP, small heterodimer partner; AMPK, 5’-AMP-activated protein kinase; ERK, Extracellular signal-regulated kinase.

To explore the mechanism of the action of EPC in BA metabolism, we examined the expression of several known related genes and proteins (Figure 6). In MAFLD rats, the expression of FXR, BSEP, and CYP8B1 mRNA was significantly downregulated in the liver. Conversely, EPC dramatically reversed the downregulation of *FXR*, *BSEP*, and *CYP8B1* mRNA downregulation after HFD feeding. In addition, EPC also remarkably upregulated *CYP7A1*, *CYP27A1*, and *NTCP* mRNA expression downregulated *CYP7B1* and *HMGCR* expression (Figures 6A–G). At protein expression level, CYP7A1 and p-ERK are significantly increased in the liver for HFD-fed rats, and the expression of CYP7A1 is visibly reduced after administration of EPC (Figures 6H,L). In addition, EPC significantly upregulated the expression of FXR and p-AMPK and downregulated the expression of p-ERK but had no significant effect on the relative expression of SHP (Figures 6I–K). In the ileum, we also found that EPC-H upregulated the relative expression of FXR (*p* < 0.05) and FGF15 (*p* > 0.05) (Figures 6M,N).

### Screening of the binding ability of active ingredients to proteins

**FIGURE 7:**
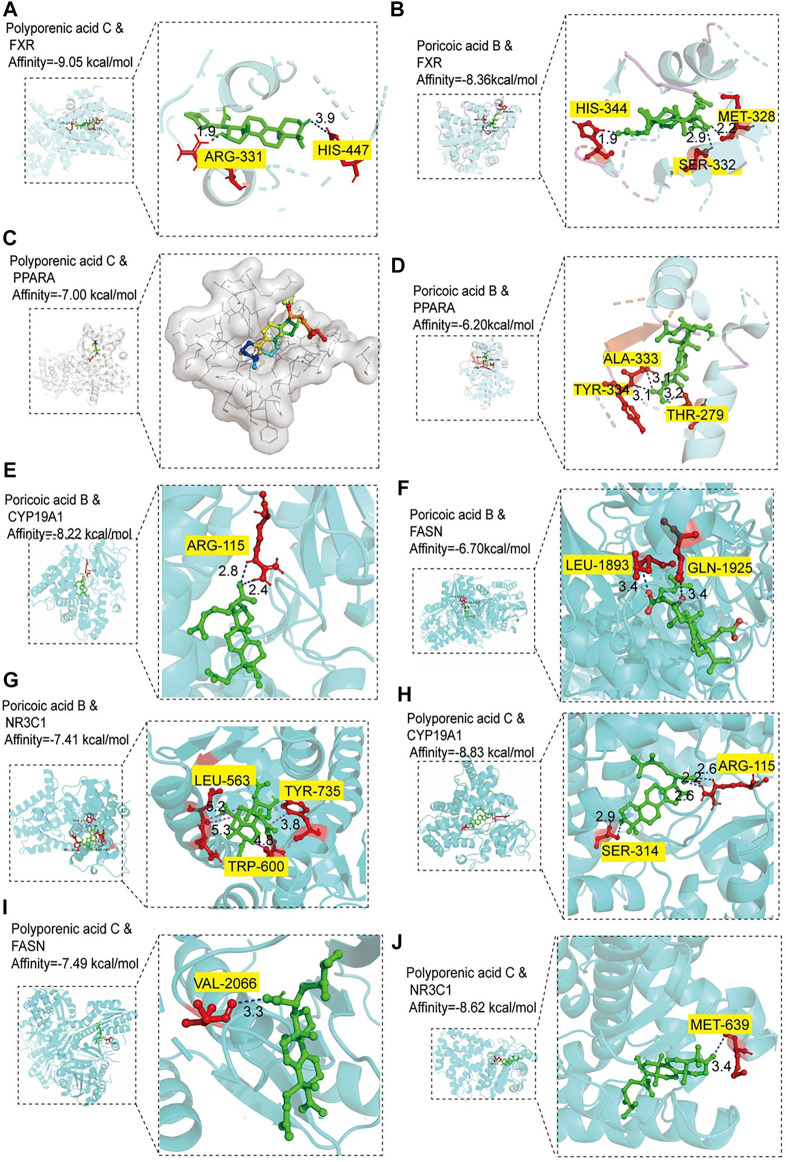
Molecular docking analysis. **(A‒B)** Molecular docking of polyporenic acid C and Poricoic acid B to farnesoid X receptor (FXR). **(C-D)** Molecular docking of polyporenic acid C and Poricoic acid B to peroxisome proliferator-activated receptor α (PPARA, PPARα). **(E‒G)** Molecular docking of poricoic acid B to aromatase (CYP19A1), fatty acid synthase (FASN), and glucocorticoid receptor (NR3C1). **(H‒J)** Molecular docking of polyporenic acid C to CYP19A1, FASN, and NR3C1. CYP19A1, aromatase; NR3C1, Glucocorticoid receptor.

To further investigate the material basis of *P. cocos* for treating MAFLD and explore its mechanism. One hundred molecular dockings were performed based on the screened critical active ingredients and targets (Supplementary Table S3). It is generally accepted that binding energy < 0 kcalmol^−1^ indicates that the ligand and receptor can react spontaneously (Meng et al., 2011) (Supplementary Table S4). The more negative the Vina score, the more stable the compound binds to the protein (Supplementary Figure S2). FXR and PPARA have excellent binding activity with polyporenic acid C and poricoic acid B (Figures 7A–D). The binding energies were -9.05 kcal/mol, −8.36 kcal/mol, −7.0 kcal/mol, −6.20 kcal/mol. A binding energy ≤ −8.0 kcal/mol indicates a robust critical activity between the ligand and the receptor (Clyne et al., 2020). In addition, we also docked FXR and PPARA upstream and downstream proteins, and the results as shown in Figures 7E–J. Poricoic acid B formed two H-bonds with CYP19A1 ARG 115 (Score = −8.22 kcal/mol). Polyporenic acid C began four H-bonds with CYP19A1, SER 314, and ARG 115 (Score = −8.83 kcal/mol) and one H-bond with NR3C1 MET 639 (Score = −8.62 kcal/mol).

### Identification of poricoic acid B and polyporenic acid C

**FIGURE 8:**
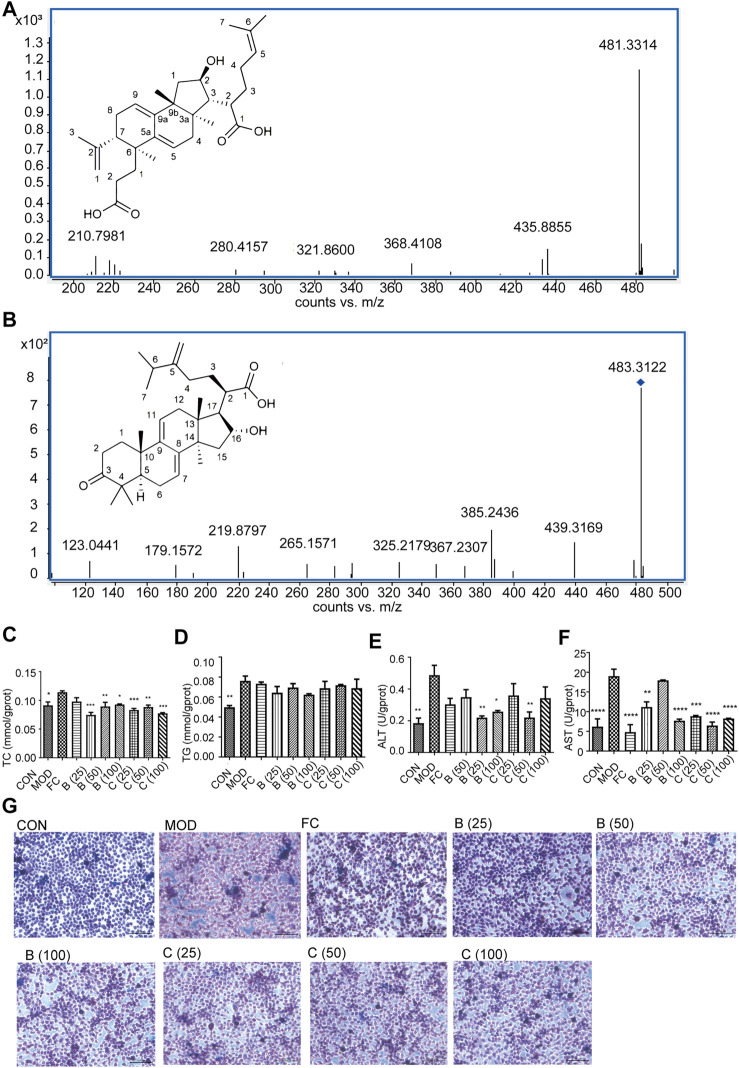
Identification of poricoic acid B and polyporenic acid C. **(A)** Characteristic ion peak and 2D structure of poricoic acid B. **(B)** Characteristic ion peak and 2D structure of polyporenic acid C. **(C‐F)** Cell levels of TC, TG, ALT, AST. **(G)** Fat accumulation in cells were evaluated under a light microscope (×200) after Oil Red O staining dose (25, 50, and 100 μmol/L) of poricoic acid B group (B (25), B (50), and B (100)), dose (25, 50, and 100 μmol/L) of polyporenic acid C group (C (25), B (50), and B (100)), and FC (150 μmol/L) or separate medium (CON group). *n* = 3, data are presented as mean ± SEM. One-way analysis of variance (ANOVA) was conducted for the group comparison. *p < 0.05, **p < 0.01, ***p < 0.001, ****p < 0.0001 *vs* MOD).

Based on the molecular docking, according to the precise molecular mass and fragmentation information from previous reports, poricoic acid B and polyporenic acid C in the EPC were identified through the UPLC Q-TOF/MS. Analysis showed that the retention time of poricoic acid B in [M-H]^-^ mode was 44.6758 min; Primary mass spectrum (m/s) was 483.312; Secondary ion fragments (ms/ms) were 467.2808, 421.3058, and 439.3169; And mass error was −4.58 ppm (Figure 8A). The retention time of polyporenic acid C in [M-H]^-^ mode was 49.8359 min; The primary mass spectrum (m/s) was 481.3351; The secondary ion fragments (ms/ms) were 421.3074, 271.1652, and 435.8855; And mass error was −5.73 ppm (Figure 8B).

### Poricoic acid B and polyporenic acid C reduced the level of L-02 adipocytes

Finally, Network pharmacology combined with UPLC Q-TOF/MS analysis showed that poricoic acid B and polyporenic acid C are the higher contributing compounds in treating MAFLD by EPC (Figures 1, 8). Besides, it is known from the literature that the content of poricoic acid B in *P. cocos* is ∼0.3578%–0.8619%, and the content of polyporenic acid C is ∼0.2369%–0.669% (Fu et al., 2018). In this study, we identified poricoic acid B and polyporenic acid C as 6.7% and 28.1% of EPC, respectively. Pharmacological studies showed that porcine acid B has a strong anti-inflammatory effect (Zhang et al., 2021). Polyporenic acid C potentiates insulin and induces differentiation of ST-13 preadipocytes (Yang et al., 2021). Based on the above findings, we chose poricoic acid B and polyporenic acid C to determine whether triterpenic acid could alleviate the damage to L-02 hepatocytes in a 5% fatty emulsion. After treatment with poricoic acid B and polyporenic acid C, we observed a reduction in intracellular lipid droplets (Figure 8G) and a significant decrease in TC, TG, ALT, and AST levels compared with that in the MOD group (Figures 8C–F).

## Discussion

Currently, exercise and dietary intraventures remain the key treatment recommendations for patients with MAFLD and its progressive stages (Pang et al., 2022). However, this can be a challenging lifestyle for patients owing to social, mental, physical, and phylogenetic reasons (Worm, 2020). Therefore, it is imperative to find pharmacological treatments for MAFLD. In China, TCM has taken a tremendous role in the prevention and treatment of MAFLD because of its unique theory, pathogenesis, diagnosis, and treatment of MAFLD, such as the theory of “Hepatic and Spleen Co-treatment” and the system of hepatic-based metabolic balance regulation (Yao et al., 2016; Ding et al., 2019). In recent years, there has been a rapidly growing amount of evidence that herbal medicines have beneficial effects in treating MAFLD, such as Pu-erh tea and mulberry leaves (Huang et al., 2019; Zhong et al., 2020). To elucidate the relationship between *P. cocos* on MAFLD, we used the TCM network pharmacology analysis system to establish the gene co-association between *P. cocos* and MAFLD. UPLC Q-TOF/MS combined with *in vitro* and *in vivo* experiments showed that *P. cocos* triterpenes were the characteristic substances of *P. cocos* for treating MAFLD.

MAFLD is initiated by excessive intake of nutrients. Although most fat is consumed through muscle oxidation, a portion is stored in adipose tissue (Lan et al., 2022). The fat stored in iWAT is further converted to fatty acids through lipolysis. Overconsumption of fatty acids leads to the accumulation of abundant TG in hepatocytes and induces hepatic steatosis (Blüher, 2013). In the present study, EPC reduced HFD-induced hepatic lipid deposition and promoted the browning of eWAT, iWAT, and pWAT white fat. The SREBP transcription factor performs an essential function within lipid metabolism and has three isoforms, SREBP-1a, SREBP-1c, and SREBP-2 (Wu et al., 2019). The *SREBP-1c* high expression can promote fatty acid synthesis into TC, leading to hepatic lipid deposition. HMGCR is the rate-limiting enzyme for TC synthesis, and the *HMGCR* gene also regulates the expression of SREBP-2 at the transcriptional level (Wu et al., 2019). Additionally, HFD and excess energy intake have been shown to reduce (Wu et al., 2019)AMPK activity, thereby increasing the expression of lipid synthesis-related genes, including *ACC*, *SREBP-1c*, and *HMGCR*, ultimately leading to increased synthesis of fatty acids, TG and TC (Chen et al., 2018). In our study, EPC inhibited the expression of *FASN*, *SREBP-1c, and HMGCR* and promoted the expression of p-AMPKα and PPARα. The above suggested that EPC may reduce hepatic fat accumulation and steatosis and inhibit spontaneous fat synthesis and fatty acid uptake while accelerating fatty acid β-oxidation.

Persistent inflammation within the liver is thought to be a key cause of further MAFLD progression, such as inhibition of NF-κB and JNK activation can reduce inflammation and thus protect the liver (Lan et al., 2022). In our research, EPC inhibited the expression of p-NF-κB, p-ERK1/2, and p-JNK in liver tissue and decreased the secretion of the pro-inflammatory cytokines IL-6 and IL-1β under metabolic stress. Furthermore, disturbances in BA metabolism occur during the progression of MAFLD when hepatic BA production is increased or metabolism is slowed down, resulting in BA stasis and thus inducing inflammation (Jiao et al., 2022). In the clinic, in patients with MAFLD, the content of primary and free BAs increased, while that of secondary and conjugated BAs decreased (Michelotti et al., 2013; Zhu et al., 2016; Jiao et al., 2022). In this study, EPC-H reduced the fecal-free BAs induced by HFD by 16.36%, and fecal concentrations of conjugated BAs were elevated. In addition, higher levels of CA, LCA, and DCA were observed in the feces of HFD-induced MAFLD rats (Michelotti et al., 2013; Zhu et al., 2016; Jiao et al., 2022); In our study, EPC not only reduced TBA and IBIL levels in rat liver but also decreased CA, DCA, and LCA levels in feces. Suggested that EPC inhibits the development of MAFLD by suppressing the release of hepatic inflammatory factors and an improved BA metabolism.

PPARa signaling pathway participates in the control of BA metabolism and lipid metabolism (Michelotti et al., 2013; Lan et al., 2022). FXR is an upstream protein expressed in the SHP and PPARα signaling pathways during MAFLD disease progression (Li et al., 2013). Also, FXR inhibits the expression of SREBP-1c by synergizing with SHP, thereby suppressing the expression of fatty acid synthase, acetyl coenzyme A carboxylase, and other key enzymes of fatty acid ab initio synthesis, and ultimately inhibiting hepatic TG synthesis. In addition, FXR activation can negatively feedback and inhibit the expression of CYP7A1 and CYP8B1, the rate-limiting enzymes of BA synthesis, thereby reducing BA synthesis and lipid absorption (Li et al., 2022; Zeng et al., 2016). In our study, EPC remarkably increased the expression of FXR and PPARα. Interestingly, EPC also substantially reduced CYP7A1 and SREBP-1c of hepatic expression. FXR agonists CDCA and GW4064 cause upregulation of PPARα expression, mainly through FXR binding to the PPARα promoter region, enhancing the expression of the PPARα target gene *CPT1*, promoting fatty acid β-oxidation, and reducing hepatic TG accumulation (Miyata et al., 2014). Furthermore, the regulatory effect of PPARα on BA metabolism is being reported extensively (Mencarelli et al., 2013; Xie et al., 2019). It has been shown that PPARα can be activated by affecting the expression of CYP7A1, NTCP, and BSEP, thereby reducing the synthesis of BAs. FXR and PPARα regulate energy homeostasis in the hepatic mainly through the activation of PPARα to promote fatty acid oxidation and through the activation of FXR to control BA homeostasis (Xie et al., 2019).

## Conclusion

**FIGURE 9:**
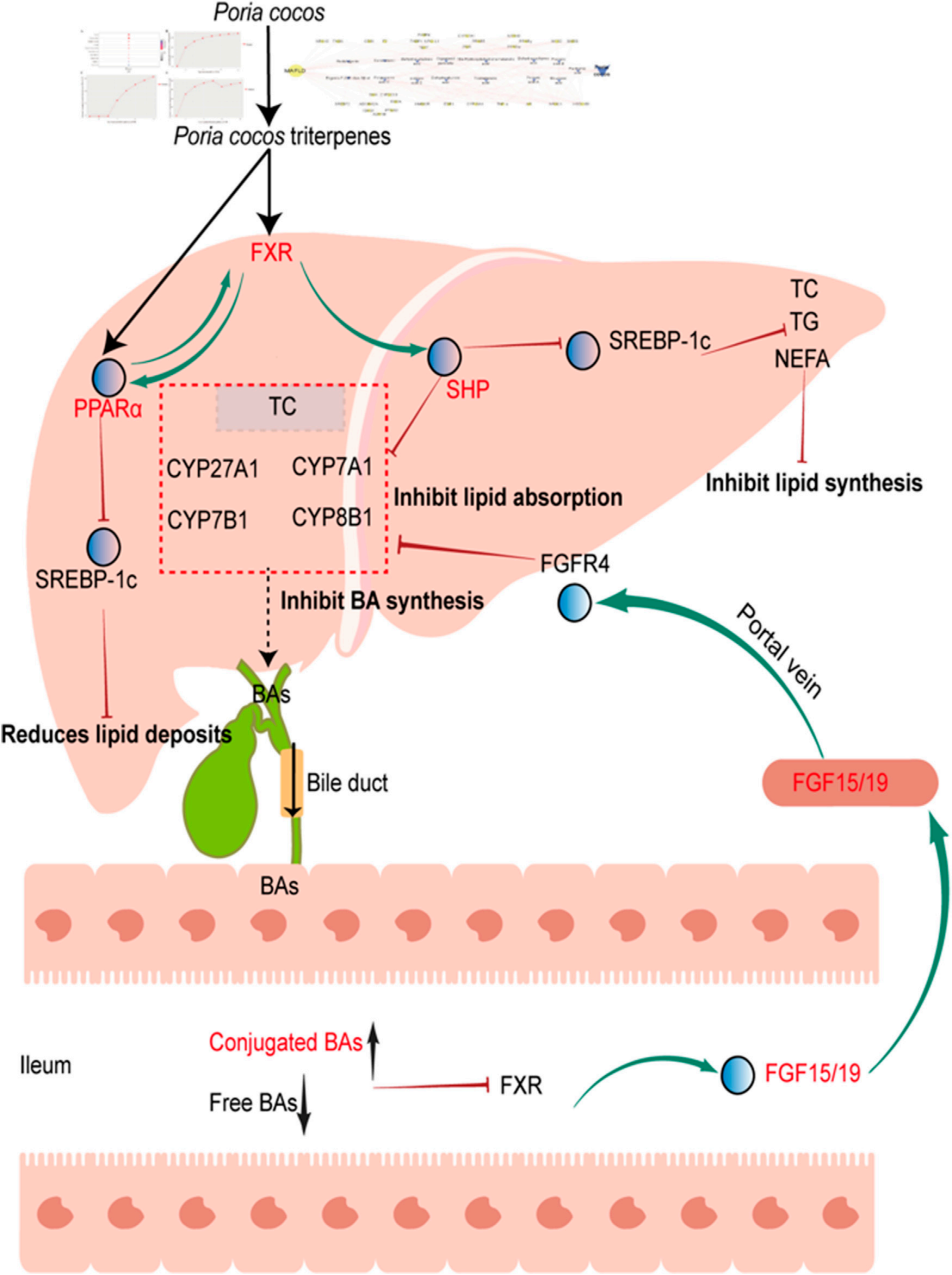
Proposed mechanism for *P. cocos* on MAFLD. *Poria cocos* activation of hepatic FXR, which can activate PPARα, inhibit SREBP-1c, reduces lipid deposits. Activation of hepatic FXR, which can activate SHP, inhibit SREBP-1c, reduce TC, TG, NEFA levels and inhibit lipid synthesis. Activation of hepatic FXR, which can activate SHP, reduce CYP7A1 expression and inhibit BA synthesis and lipid absorption; In addition, decreased free bile acids and increased bound bile acids in the intestine can inhibit FXR expression, promote FGF15/19 expression, and reduced bile acid absorption by binding to FGFR4 and inhibiting CYP7A1 expression. FGF15/19, fibroblast growth factor FGF15/19. FGFR4, fibroblast growth factor receptor 4.

In summary, EPC alleviated HFD-induced MAFLD through the regulation of lipid homeostasis and BA metabolism. *P. cocos* triterpenes, such as poricoic acid B and polyporenic acid C, are the characteristic substances of *P. cocos* for the treatment of MAFLD, and its mechanism may be *via* the FXR/PPARα-SREBPs signaling pathway (Figure 9).

## Data Availability

The raw data supporting the conclusions of this article will be made available by the authors, without undue reservation.

## Abbreviations

CYP7A1, cholesterol 7α-hydroxylase; FXR, farnesoid X receptor; FGF 15, fibroblast growth factor15; HFD, high-fat diet; MAFLD, metabolic dysfunction-associated fatty liver disease; NEFA, nonesterified free fatty acids; PPARα, peroxisome proliferator-activated receptor α; SREBP-1c, sterol regulatory element binding protein 1c; TCM, traditional Chinese medicine; TC, cholesterol; TBA, total bile acid; UHPLC Q-Trap/MS, ultra-high performance liquid chromatography coupled with hybrid triple quadrupole linear ion trap mass spectrometer; UPLC Q-TOF/MS: ultraperformance liquid chromatography quadrupole time-of-flight mass spectrometry.
