# Corrigendum: Effects of *Poria cocos* extract on metabolic dysfunction-associated fatty liver disease via the FXR/PPARα-SREBPs pathway

**DOI:** 10.3389/fphar.2023.1172950

**Published:** 2023-05-19

**Authors:** Jinbiao He, Yu Yang, Fan Zhang, Yanjuan Li, Xiaosi Li, Xuemei Pu, Xudong He, Mei Zhang, Xinxing Yang, Qiuman Yu, Yan Qi, Xuefang Li, Jie Yu

**Affiliations:** College of Pharmaceutical Science, School of Clinical Medicine, Yunnan Key Laboratory of Southern Medicinal Utilization, Yunnan University of Chinese Medicine, Kunming, China

**Keywords:** MAFLD (metabolic-associated fatty liver disease), *Poria cocos* (Schw.) Wolf., bile acid metabolism, FXR/PPARα-SREBP pathway, lipid homeostasis, UPLC Q-TOF/MS

In the published article, there was an error in the legend for **Figure 2** as published. In the legend for **Figure 2**, “(N) Brown adipose tissue (BAT).” is a duplicate and needs to be deleted because the BAT is explained in the legend for **Figure 2I**. The corrected legend appears below.

“FIGURE 2 | EPC ameliorated MAFLD in rats. **(A)** Body weight (BW). **(B)** BW gain. **(C–E)** Organ wet weight. **(F)** Inguinal white adipose tissue (iWAT). **(G)** Perirenal white adipose tissue (pWAT). **(H)** Epididymis white adipose tissue (eWAT). (**I**) Brown adipose tissue (BAT). **(J)** iWAT/BW ratio. **(K)** pWAT/BW ratio; **(L)** eWAT/BW rati. (**M**) BAT/BW ratio. **(N)** Representative rat liver images of hematoxylin and eosin (H and E) and Oil Red O staining per group (X200). **(O)** Representative iWAT, pWAT, eWAT, BAT. One-way analysis of variance (ANOVA) was conducted for the group comparison. *n* = 8, data are presented as mean ± SEM.*p < 0.05, **p < 0.01, ***p < 0.001 vs*.* MOD group. EPC, *P. cocos* ethanol extract; CON, normal diet control group; MOD, high-fat diet group; FC, Fenofibrate capsules; EPC-L, low-dose *P. cocos* ethanol extract; EPC-H, high-dose *P. cocos* ethanol extract.].

Furthermore, there was an error in Figure 6P as published. The authors apologize for uploading the ERK protein image in Figure 6 incorrectly, with image of p-JNK, in this article. Furthermore, P-ERK should be p-ERK in Figure 6P. The corrected Figure 6 appears below.

**FIGURE 6:**
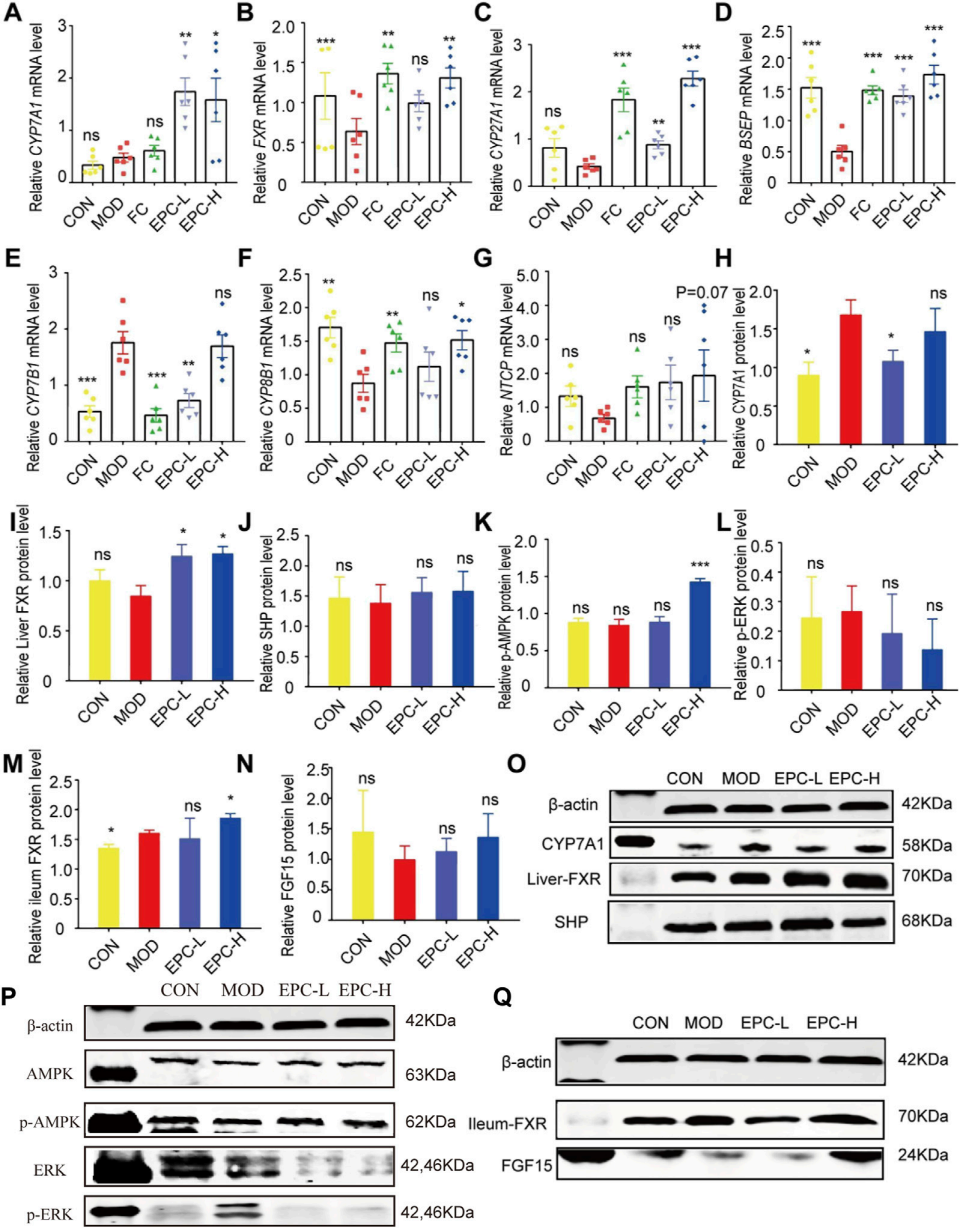
EPC ameliorated MAFLD formation in rats by regulating BA metabolism. **(A–G)** Relative expression of CYP7A1, FXR, CYP27A1, BSEP, CYP7B1, CYP8B1, NTCP mRNA in liver, n = 6; **(H–L)** Relative expression of protein CYP7A1, FXR, SHP, p-AMPK, and p-ERK in the liver, n = 4; **(M–N)** Relative expression of protein FXR and FGF15 in the ileum, n = 4. **(O–P)** Representative immunoblotting images of CYP7A1, FXR, SHP, p-AMPK,and p-ERK in the liver. **(Q)** Representative immunoblotting images of FXR and FGF15 in the ileum. Data are presented as mean ± SEM. One-way analysis of variance (ANOVA) was conducted for the group comparison. *p < 0.05, **p < 0.01, ***p < 0.001 vs. MOD group. CYP7A1, cholesterol 7α-hydroxylase; FXR, farnesoid X receptor; CYP27A1, sterol 27-hydroxylase; BSEP, bile salt export protein; CYP7B1, oxysterol 7α-hydroxylase; CYP8B1, sterol 12αhydroxylase; NTCP, Na + -taurocholate co-transporting polypeptides; SHP, small heterodimer partner; AMPK, 5′-AMP-activated protein kinase; ERK, Extracellular signal-regulated kinase.

