# Supplementary material for: Effects of Poria cocos extract on metabolic dysfunction-associated fatty liver disease via the FXR/PPARα-SREBPs pathway
Source: Front Pharmacol. 2022 Oct 5;13:1007274. doi: 10.3389/fphar.2022.1007274 (PMC9581278; doi:10.3389/fphar.2022.1007274)
Supplement: Supplementary file 1 [file DataSheet1.pdf]

Supplementary Material

Supplementary Figure 1

(A) Venn diagram of targets in *P. cocos* and metabolic dysfunction-associated fatty liver disease (MAFLD);

(B) Protein–protein interaction network of the potential targets;

(C) Gene ontology (GO) enrichment analysis for key targets;

(D) Organ localization result. The square represents the core target, V shape represents the system corresponding to the positioning organ, Ellipses represent localized tissues and organs.

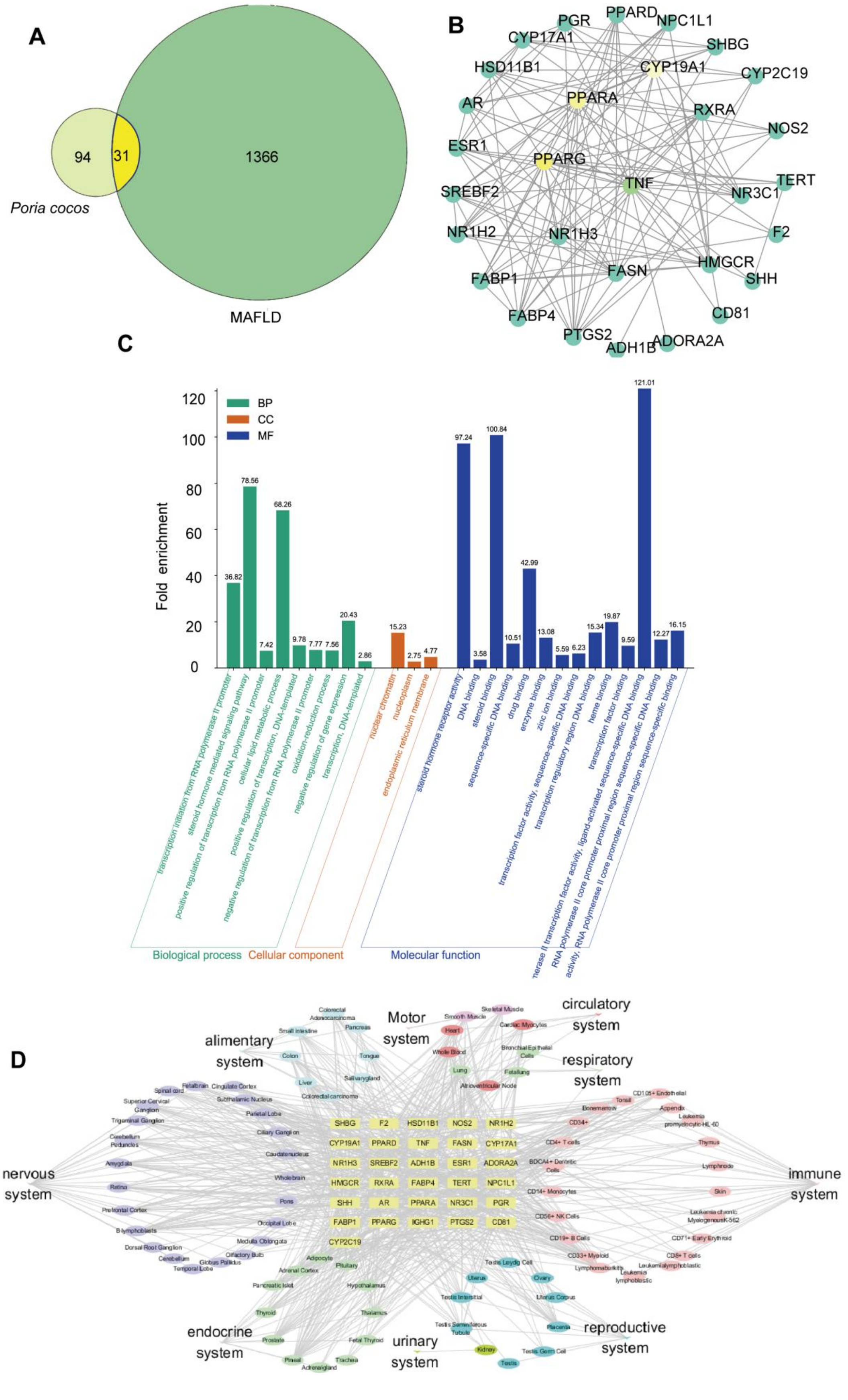
A
94
31
1366
Poria cocos
MAFLD
B
PGR
PPARD
NPC1L1
CYP17A1
SHBG
HSD11B1
CYP19A1
CYP2C19
AR
PPARA
RXRA
NOS2
ESR1
PPARG
TNF
TERT
SREBF2
NR3C1
NR1H2
NR1H3
F2
HMGCR
FASN
FABP1
SHH
CD81
FABP4
PTGS2
ADH1B
ADORA2A
C
BP
CC
MF
Fold enrichment
36.82
78.56
7.42
68.26
9.78
7.77
7.56
20.43
2.86
15.23
2.75
4.77
97.24
3.58
100.84
10.51
42.99
13.08
5.59
6.23
15.34
19.87
9.59
121.01
12.27
16.15
transcription initiation from RNA polymerase II promoter
steroid hormone mediated signaling pathway
positive regulation of transcription from RNA polymerase II promoter
cellular lipid metabolic process
positive regulation of transcription, DNA-templated
negative regulation of transcription from RNA polymerase II promoter
oxidation-reduction process
negative regulation of gene expression
transcription, DNA-templated
nuclear chromatin
nucleoplasm
endoplasmic reticulum membrane
steroid hormone receptor activity
DNA binding
steroid binding
sequence-specific DNA binding
drug binding
enzyme binding
zinc ion binding
transcription factor activity, sequence-specific DNA binding
transcription regulatory region DNA binding
heme binding
transcription factor binding
RNA polymerase II transcription factor activity, ligand-activated sequence-specific DNA binding
RNA polymerase II core promoter proximal region sequence-specific DNA binding
activity, RNA polymerase II core promoter proximal region sequence-specific binding
Biological process
Cellular component
Molecular function
D
alimentary system
Motor system
circulatory system
respiratory system
nervous system
immune system
endocrine system
urinary system
reproductive system
SHBG
F2
HSD11B1
NOS2
NR1H2
CYP19A1
PPARD
TNF
FASN
CYP17A1
NR1H3
SREBF2
ADH1B
ESR1
ADORA2A
HMGCR
RXRA
FABP4
TERT
NPC1L1
SHH
AR
PPARA
NR3C1
PGR
FABP1
PPARG
IGHG1
PTGS2
CD81
CYP2C19

Supplementary Figure 2 Molecular docking heat map

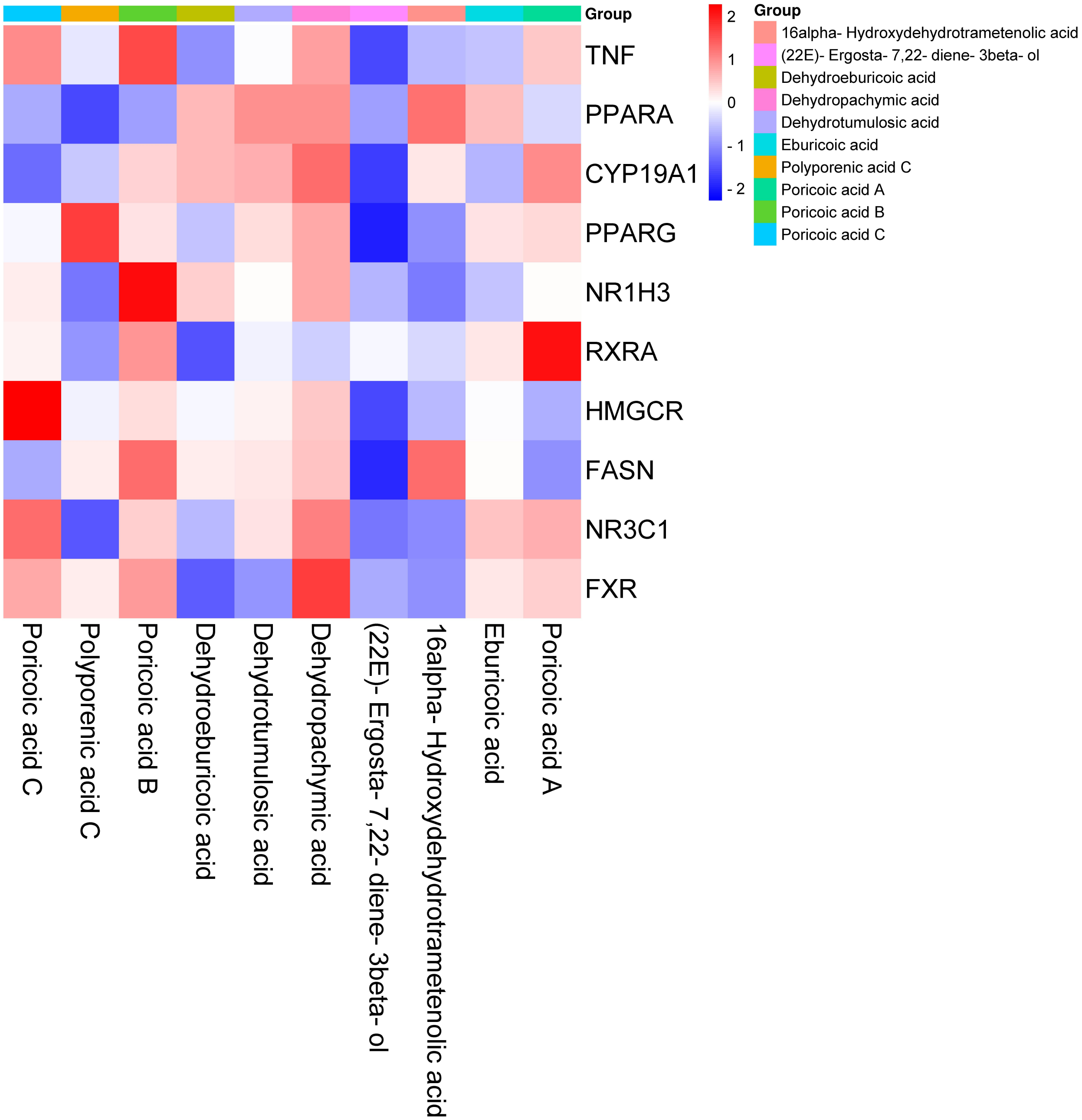

Supplementary table 1

Table S1. Primers used for RT-qPCR analysis

mRNA	Forwad	Rerverse
NR3C1	TGTTAGGTGGGCGTCAAGTGATTG	CTGTAGCAGGGTCATTTGGTCATCC
CYP19A1	GAGAGTCTGGATCAGTGGAGAGGAG	CTTGCTGCCGAATCTGGAGATGTAG
FASN	GTGTGGTAGGCTTGGTGAACTGTC	GTGAGATGTGCTGCTGAGGTTGG
HMGCR	GACCAACCTTCTACCTCAGCAAGC	GGACAACTCACCAGCCATCACAG
RXRα	CTCCTTCTCCCACCGCTCCATAG	CTCCGTTAGCACCCTGTCAAAGATG
SREBP-1c	TCCACCATCGGCACCCACTG	GGCACTGGCTCCTCTTTGATTCC
CYP7A1	AGAGGCTGCTTTCATTGCTTCAGG	TCGCACCAATGTGAATCTGGCTAG
CYP8B1	TTTCTGAGGGAGCGAGGGATGG	CCTTCATGGCGTCCTGATGCTTC
CYP27A1	TCGCACCAATGTGAATCTGGCTAG	CTTCCACTGCTCCATGCTGTCTC
CYP7B1	GGGCCTCTCTAGCAAACACCATTC	CGTCACGCAGGACTTCCATAGC
NTCP	ACCTACAAAGCTGCTGCAACTGAG	GGAAGGACCAGGTTGGAGAGGAG
BSEP	CGGCAACGCTCCAAGTCTCAG	GGGGCAGGTTCAACTTCTTCCAC
FXR	AGGATAGAGAGGCAGTGGAGAAGC	AGCGTGGTGATGGTTGAATGTCC
SCD	TGTCAAAGAGAAGGGCGGAAAGC	CAGGATGAAGCACATGAGCAGGAG

Supplementary table 2

Table S2. *P. cocos* active ingredients

MOL ID	Chemical name	OB	DL
MOL000273	(2R)-2-[(3S,5R,10S,13R,14R,16R,17R)-3,16-dihydroxy-4,4,10,13,14-pentamethyl-2,3,5,6,12,15,16,17-octahydro-1H-cyclopenta[a]phenanthren-17-yl]-6-methylhept-5-enoic acid	30.93	0.81
MOL000275	trametenolic acid	38.71	0.8
MOL000276	7,9(11)-dehydropachymic acid	35.11	0.81
MOL000279	Cerevisterol	37.96	0.77
MOL000280	(2R)-2-[(3S,5R,10S,13R,14R,16R,17R)-3,16-dihydroxy-4,4,10,13,14-pentamethyl-2,3,5,6,12,15,16,17-octahydro-1H-cyclopenta[a]phenanthren-17-yl]-5-isopropyl-hex-5-enoic acid	31.07	0.82
MOL000282	ergosta-7,22E-dien-3beta-ol	43.51	0.72
MOL000283	Ergosterol peroxide	40.36	0.81
MOL000285	(2R)-2-[(5R,10S,13R,14R,16R,17R)-16-hydroxy-3-keto-4,4,10,13,14-pentamethyl-1,2,5,6,12,15,16,17-octahydrocyclopenta[a]phenanthren-17-yl]-5-isopropyl-hex-5-enoic acid	38.26	0.82
MOL000287	3beta-Hydroxy-24-methylene-8-lanostene-21-oic acid	38.7	0.81
MOL000289	pachymic acid	33.63	0.81
MOL000290	Poricoic acid A	30.61	0.76
MOL000291	Poricoic acid B	30.52	0.75
MOL000292	poricoic acid C	38.15	0.75
MOL000296	hederagenin	36.91	0.75
MOL000300	dehydroeburicoic acid	44.17	0.83

Supplementary table 3

Table S3. Topological parameters of target nodes for *P. cocos* treatment of MAFLD

Name	N	BC	CC	EC	LAC
TNF	23.00	0.31	0.81	3.00	6.35
PPARA	16.00	0.12	0.69	2.00	6.13
PPARG	15.00	0.05	0.66	3.00	6.13
CYP19A1	13.00	0.05	0.64	2.00	6.15
RXRA	13.00	0.02	0.63	3.00	7.08
FASN	13.00	0.04	0.63	3.00	7.08
NR1H3	12.00	0.01	0.60	3.00	8.00
HMGCR	11.00	0.02	0.60	3.00	6.91
NR3C1	10.00	0.01	0.58	3.00	5.80

Supplementary table 4

Table S4: Molecular docking of the main active ingredients of *P. cocos* and core proteins.

**Core active ingredient**	**Docking Binding Energy/ kcal.mol－1**									
	TNF	PPARA	CYP19A1	PPARG	NR1H3	RXRA	HMGCR	FASN	NR3C1	FXR
Poricoic acid C	-3.86	-6.1	-9.42	-3.38	-9.51	-5.21	-5.9	-8.15	-6.85	-8.49
Polyporenic acid C	-4.32	-7	-8.83		-12.08	-5.93	-6.83	-7.49	-8.62	-9.05
Poricoic acid B	-3.62	-6.2	-8.22	-2.76	-5.89	-4.64	-6.67	-6.7	-7.41	-8.36
Dehydroeburicoic acid	-4.63	-4.66	-8.03	-4.39	-9.04	-6.34	-6.8	-7.49	-8.07	-10.54
Dehydrotumulosic acid	-4.25	-4.29	-7.98	-2.63	-9.78	-5.37	-6.73	-7.47	-7.52	-10.08
Dehydropachymic acid	-3.93	-4.29	-7.56	-1.66	-8.42	-5.59	-6.6	-7.25	-6.96	-7.59
(22E)-Ergosta-7,22-diene-3beta-ol	-4.88	-6.16	-9.73	-7.54	-11.01	-5.33	-7.4	-8.96	-8.46	-9.92
16alpha-Hydroxydehydrotrametenolic acid	-4.49	-3.98	-8.34	-5.34	-11.99	-5.52	-7.01	-6.68	-8.32	-10.14
Eburicoic acid	-4.46	-4.72	-8.97	-2.77	-10.81	-5.14	-6.79	-7.61	-7.33	-9.01

Poricoic acid A	-4.06	-5.66	-7.75	-2.58	-9.83	-3.84	-7.05	-8.32	-7.23	-8.80
